# Exosomal PD-L1 and N-cadherin predict pulmonary metastasis progression for osteosarcoma patients

**DOI:** 10.1186/s12951-020-00710-6

**Published:** 2020-10-22

**Authors:** Jun Wang, Hongliang Zhang, Xin Sun, Xiaofang Wang, Tingting Ren, Yi Huang, Ranxin Zhang, Bingxin Zheng, Wei Guo

**Affiliations:** 1 Peking University People’s Hospital, Musculoskeletal Tumor Center, No. 11 Xizhimen South Street, Beijing, 100044 China; 2 Beijing Key Laboratory of Bio-Characteristic Profiling for Evaluation of Rational Drug Use, International Cooperation & Joint Laboratoryof Bio-Characteristic Profiling for Evaluation of Rational Drug Use, Beijing Shijitan Hospital, Capital Medical University,, Beijing, 100038 China

**Keywords:** Exosome, programmed death-ligand 1, N-cadherin, osteosarcoma, biomarker

## Abstract

**Background:**

Recent studies indicated that exosomal programmed death-ligand 1 (PD-L1) derived from cancers could induce immunosuppression and tumor pathogenesis. However, it is unclear how exosomes influence osteosarcoma (OS) progression and whether PD-L1 also exists in serum exosomes (Sr-exosomes) of patients with osteosarcoma. We examined serum exosomes from 70 OS patients, 9 patients with benign tumors and 22 healthy donors. OS-derived exosomes were functionally evaluated in vivo and in vitro.

**Results:**

The characteristics of exosomes derived from OS patient serum and OS cell lines were confirmed by several methods. We found OS patients had a higher level of exosomal PD-L1 compared to healthy donors. Meanwhile, OS patients with pulmonary metastasis also showed a relatively higher level of exosomal PD-L1 than patients without metastasis. Next, bioinformatic analysis demonstrated that Sr-exosomes isolated from OS patients may involve in the important process of immune function and cancer pathogenesis for OS patients. Co-expression network centered with PD-L1 among Sr-exosomal differently expressed mRNA demonstrated exosomal N-cadherin had a close relationship with exosomal PD-L1 expression. Then, we confirmed higher level of Sr-exosomal N-cadherin in OS patients with pulmonary metastasis compared to ones without metastasis. Furthermore, we elucidated osteosarcoma-derived exosomes and exosomal-PD-L1 promoted the pulmonary metastasis in metastatic models. ROC (Receiver Operating Characteristic Curve) analysis showed AUC (Area Under Curve) of 0.823 for exosomal PD-L1, 0.806 for exosomal N-cadherin and 0.817 for exosomal N-cadherin/E-cadherin to distinguish OS patients with pulmonary metastasis from ones without metastasis.

**Conclusions:**

Osteosarcoma stimulates pulmonary metastasis by releasing exosomes, that carry PD-L1 and N-cadherin. Detection of exosomal PD-L1 and N-cadherin from serum of OS patients may predict pulmonary metastasis progression for OS patients. 
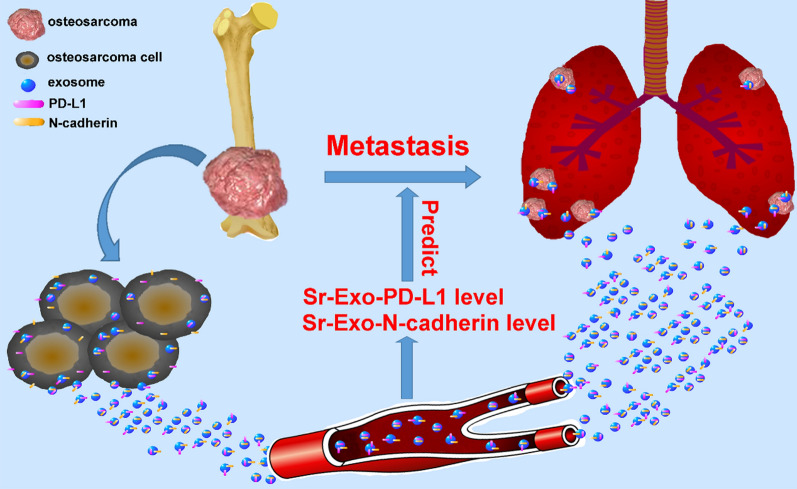

## Introduction

Exosomes are secreted as the vesicles which are enclosed by the cell membrane and their size ranges from 30 to 150 nm diameter [1]. Various types of cells such as immune cells, fibroblast and endothelial cells, release exosomes into the extracellular space and microenviroment, involving in the tumor pathogenesis [2–5]. Recent studies reported that tumor cell–derived exosomes could stimulate tumor cell growth, survival, metastasis and chemotherapy resistance [6–12]. Meanwhile, exosomes isolated from cancer cells can evade host immune surveillance, modulate tumor microenvironment and initiate the formation of premetastatic sites for the immunosuppression [13, 14]. As a major malignant bone tumor, osteosarcoma commonly occurs in children and young adults and pulmonary metastasis occurs in approximately 15–20% of patients [12]. Neo-adjuvant chemotherapy has increased the 5-year survival for patients with localized tumors to 60–80%. Patients with metastatic disease remain refractory to chemotherapy and have a 5-year survival of only 10–20% [12, 15, 16]. However, prediction of pulmonary metastasis for OS patients is a challenge for oncologists. To date, it lacks the powerful biomarker to predict the pulmonary metastasis during the follow-up. Exosomes are reported to involve in the process of pulmonary metastasis and can be utilized as a biomarker to monitor the tumor progression [17–19]. It is well established that osteosarcoma generates exosomes, which play an important role in tumor progression and metastasis [20–22].

Programmed death ligand 1 (PD-L1; B7-H1; CD274) was firstly discovered by Freeman et al. in 2000, who reported that PD-1/PD-L1 interaction resulted in inhibited TCR-mediated CD8^+^ lymphocyte activation. PD-L1 on the surface of tumor cells binds its receptor PD-1 on effector T cells, thereby suppressing their activity [23]. PD-1/PD-L1 axis and its role in promoting immune escape has resulted in great development in the therapeutical strategy and tumor pathogenesis mechanism. Recent studies suggested the presence of PD-L1 in extracellular vesicles isolated from blood samples of patients with cancer and the level of PD-L1 correlated with pathological features of these patients [24, 25]. Notably, exosomal PD-L1 derived from tumor cells can induce immunosuppression and tumor pathogenesis. Poggio et al. reported that exosomal PD-L1 from the tumor suppressed immune cell activation in the draining lymph node and furthermore, systemically introduced exosomal PD-L1 rescued growth of tumors which were unable to secrete their own [24]. Chen et al. revealed a rationale of circulating exosomal PD-L1 as a predictor for the clinical outcomes of anti-PD-1 therapy and they demonstrated the level of PD-L1 in circulating exosomes distinguished patients with metastatic melanoma from healthy donors. Based on their attractive findings, they recommended that developing exosomal PD-L1 as a blood-based biomarker could be an attractive option, considering the heterogeneity and dynamic changes of PD-L1 expression in tumours and the invasive nature of tumour biopsy [25]. Immune suppression driven by PD-1/PD-L1 inhibits the functions of T cells in a broad range of cancer types, including OS [26]. Our previous study also showed that PD-L1-associated poor prognosis in osteosarcoma may due to immune suppression, chemotherapy resistance, and metastasis-related pathways [27]. However, it is unclear whether PD-L1 also exists in serum exosomes of OS patients and how exosomes influence the osteosarcoma progression. Therefore, it is urgent to elucidate the exact effect of exosomes and exosomal PD-L1 in the process of pulmonary metastasis for OS patients.

## Methods

### Sample collection

79 patients (70 osteosarcomas and 9 benign tumors) were recruited from the bone tumor center of Peking University People’s Hospital. Patients were subjected to surgery between 2018 and 2019 at our tumor center. 22 healthy donors were included in the analysis. Fasting blood was collected in serum separator vacutainer tubes. All clinical samples were collected with written informed consent from patients and healthy donors, and the ethical approval was granted from the Committees for Ethical Review at our hospital.

### Cell lines culture

Human HOS, KHOS, U_2_OS and 143B cell lines were preserved in our laboratory. The cells were repeatedly cultured in RPMI 1640 with 10% fetal bovine serum, 100 U/mL penicillin, and 100 μg/mL streptomycin in a humidified cell incubator with an atmosphere of 5% CO_2_ at 37 °C. Cells growing at an exponential rate were used for the experiments.

### Generation of stable Rab27a and PD-L1 knockdown 143B cells

Lentiviruses containing shRNA targeting human Rab27a or PD-L1 (Hanheng Biotechnology, Shanghai, China) were transfected into 143B cells to knock down endogenous Rab27a or PD-L1. Short hairpin RNAs (shRNAs) against human Rab27a (Top strand: GATCCGCCAAGCAATTGAGATGCTTCTGGATTCAAGAGATCCAGAAGCATCTCAATTGCTTGGCTTTTTTG; Bottom strand: AATTCAAAAAAGCCAAGCAATTGAGATGCTTCTGGATCTCTTGAATCCAGAAGCATCTCAATTGCTTGGCG) or human PD-L1 (Top strand: GATCCGCGAATTACTGTGAAAGTCAATTTCAAGAGAATTGACTTTCACAGTAATTCGTTTTTTG; Bottom strand: AATTCAAAAAACGAATTACTGTGAAAGTCAATTCTCTTGAAATTGACTTTCACAGTAATTCGCG) were packaged into lentiviral particles using 293 T cells which was co-transfected with the viral packaging plasmids. Lentiviral supernatants were harvested 48 h after transfection. 143B cells were infected with filtered lentivirus and selected by 1 μg/mL puromycin.

### Quantitative PCR (qPCR)

Total RNA was isolated from 143B-WT, 143B-Rab27aKD and 143B-PD-L1KD using TRIzol Reagent (Invitrogen), and reverse transcribed into first-strand complementary DNA (cDNA) with random primer with RevertAid First Strand cDNA Synthesis Kit (ThermoFisher Scientific). The samples were then analysed in an Applied Biosystems QuantStudio 3 Real-Time PCR system. mRNAs of PD-L1 and Rab27a were quantified and GAPDH was used as an internal control.

### Exosome isolation

Cells were subsequently cultured with FBS-free DMEM medium for 72 h. Conditioned culture media was collected. Exosomes were isolated from the conditioned culture media and serum samples by four successive centrifugation steps as the following steps. Culture media and serum samples were centrifuged at 300*g* for 10 min at 4 °C to discard floating cells. Supernatants were centrifuged at 800*g* for 30 min, and 10,000*g* for 30 min to further purify it. Then 10 mL sample was diluted in 10 mL PBS and filtered by a 0.22 μm filter. After that, the samples were centrifuged at 150,000 g for 2 h at 4 °C. The supernatant was discarded, and exosome pellet was re-suspended in PBS. The samples were centrifuged at 150,000*g* 4 °C for 2 h in a last step. The final pellets of exosomes were stored at − 80 °C for the experiment.

For iodixanol density gradient centrifugation, exosomes harvested by differential centrifugation were loaded on top of a discontinuous iodixanol gradient (5%, 10%, 20% and 40%, made by diluting 60% OptiPrep aqueous iodixanol with 0.25 M sucrose in 10 mM Tris) and centrifuged at 100,000*g* for 18 h at 4 °C (Beckman Coulter). The exosomes distribute at the density range between 1.13 and 1.19 g/mL. The exosomes were further pelleted by ultracentrifugation at 100,000*g* for 2 h at 4 °C [24, 25].

### Characterization of purified exosomes

For transmission electron microscopy (TEM) analysis, the exosome pellet resuspending solution was loaded onto formvar/carbon-coated EM grids, fixed with 2% paraformaldehyde (PFA), and stained with 1% aqueous uranyl acetate, then dried at room temperature. The samples were examined using an device TEM. For nanoparticle tracking analysis (NTA), vesicle enriched suspension with concentrations between 1 × 10^7^/mL and 1 × 10^9^/mL was examined using the ZetaView PMX 110 (Particle Metrix, Meerbusch, Germany) equipped with a 405 nm laser to determine the size and quantity of particles. A video of 60-s duration was taken with a frame rate of 30 frames/sec, and particle movement was analyzed using NTA software.

### Western blot analysis

The concentration of exosomal total protein was quantified by the bicinchoninic acid (BCA) assay (Thermo Fisher Inc.) using bovine serum albumin (BSA) as standard. The pellets were also dissolved in 200 μL RIPA buffer for protein assay. The samples were individually homogenized in 5 mM Tris–HCI (4 mM EDTA, pH 7.4, containing 1 M pepstatin, 100 M leupeptin, 100 M phenylmethyl sulfonylfluoride, and 10 g/mL aprotinin) and cleared by centrifugation at 14,000*g* for 10 min at 4 °C. Approximately 100 μg of protein were run on a discontinuous SDS-PAGE gel and transferred to a nitrocellulose membrane. The membranes were blocked with 5% skim milk in TBS containing 0.05% Tween 20 and were incubated with the following primary antibodies: (1) Mouse anti-CD9 (sc-13118, Santa Cruz), (2) Mouse anti-CD63 (sc-5275, Santa Cruz), (3) Mouse anti-TSG101 (sc-7964, Santa Cruz), (4) Mouse anti-Alix (sc-53540, Santa Cruz), (5) Mouse anti-calnexin (10,427–2-AP, Promega, Madison), (6) Mouse anti-PD-L1 (sc-293425, Santa Cruz), (7) Rabbit monoclonal anti-E-cadherin (ab194982), (8) Rabbit polyclonal anti-N-cadherin (ab18203) and (9) Mouse anti-GAPDH (sc-47724, Santa Cruz, CA, USA). The optical density (OD) of the signals was quantified and expressed as the ratio of the tested proteins to GAPDH for analysis of protein in cells and to ALIX for analysis of protein in exosomes.

### Comparision of RNA and protein quantification in Sr-exosomes between patients with OS and healthy donors

Total RNA was isolated from serum exosomes of OS patients and healthy donors using TRIzol Reagent (Invitrogen) and the concentration of exosomal RNA in the serum was analyzed between six healthy donors and six OS patients. Meanwhile, the concentration of exosomal total protein was quantified by the bicinchoninic acid (BCA) assay (Thermo Fisher Inc.) using bovine serum albumin (BSA) as standard. The concentration of exosomal total protein in the serum was analyzed between six healthy donors and six OS patients.

### Immunohistochemistry analysis

Paraffin sections were incubated with the corresponding antibodies and stained with nonimmune serum in PBS instead of the primary antibody as the negative control. The immunostaining assessment was conducted by two independent pathologists without any previous knowledge of the clinical characteristics and outcomes [27]. The primary antibodies were as follows: (1) Mouse anti-PD-L1 (sc-293425, Santa Cruz), (2) Rabbit monoclonal anti-E-cadherin (ab194982), (3) Rabbit polyclonal anti-N-cadherin (ab18203).

### Immunogold labeling

Fixed specimens at an optimal concentration were placed onto a 400 mesh carbon/formvar coated grids and allowed to absorb to the formvar for a minimum of 1 min. The grids were placed into a blocking buffer for a block/permeabilization step for one hour. Without rinsing, the grids were immediately placed into the primary antibody at the appropriate dilution overnight at 4 °C (Mouse anti-PD-L1, sc-293425, Santa Cruz). As controls, some of the grids were not exposed to the primary antibody. The following day, all the grids were rinsed with PBS then floated on drops of the appropriate secondary antibody attached with 10 nm gold particles (AURION, Hatfield, PA) for two hours at room temperature. Grids were rinsed with PBS and were placed in 2.5% Glutaraldehyde in 0.1 M Phosphate buffer for 15 min. After rinsing in PBS and distilled water, the grids were allowed to dry and stained for contrast using uranyl acetate. The samples were viewed with a Tecnai Bio Twin transmission electron microscope (FEI, Hillsboro, OR) and images were taken with an AMT CCD Camera (Advanced Microscopy Techniques, Danvers, MA).

### Bioinformatics analysis of Sr-exosomes from 20 OS patients and 6 healthy donors

Exosomal RNA was extracted using RNeasy^®^ Mini kit (Qiagen, cat. No. 217004) according to the manufacturer’s instructions. RNA quality was assessed by using UV 260/280. The pellets were also dissolved in 1 mL Trizol LS reagent (Invitrogen) for RNA isolation. Then, we used NGS to get sequencing data of mRNA in different Sr-exosomes from 20 OS patients and 6 healthy donors. These sequencing data were analyzed by bioinformatics methods. Differently expressed genes were identified based on RVM t test and false discovery rate (FDR) analysis. Differently expressed genes with at least 1.2-fold change in either direction with *p* < 0.05 were considered to be up or downregulated. Based on the Kyoto Encyclopedia of Genes and Genomes (KEGG) database, significantly changed pathways were identified. GO analysis was used to organize differently expressed genes into hierarchical categories.

### Exosome labeling and osteosarcoma cell uptake in vitro

To examine whether Sr-exosomes derived from OS patients can be taken up by osteosarcoma cell lines 143B and U_2_OS, PKH26 (Sigma-Aldrich, PKH26GL) was used to label exosomes. Exosomes or PBS were stained with PKH26 dye in 400 μL of Diluent C for 4 min at room temperature. Then an equal volume of 1% BSA were used to stop the labeling reaction, after which they were washed with PBS and ultracentrifuged again. The labeled exosomes or PBS were respectively incubated with 143B and U_2_OS cells with complete medium for four hours at 37 °C in an atmosphere of 5% CO_2_. Then cells were washed three times in PBS to eliminate the influence of serum exosomes. The cell nuclei was counterstained with DAPI for 8 min and cell membrane was counterstained with CFSE (Sigma-Aldrich) for 5 min. The uptake of labeled exosomes by 143B and U_2_OS cells was assessed using an inverted confocal microscope.

### Cell counting kit 8 (CCK-8) assay and transwell assay

For the assessment of serum exosomes derived from OS patients on the cell proliferation of 143B and U_2_OS, cells were seeded at a density of 2000 cells per well in 96-well plates. The cells were treated with different exosome concentrations of 10, 20, 30, 40 μg /ml for 24, 48 and 72 h. Cell proliferation was analyzed using Cell Counting Kit 8 (Dojindo, Kumamoto, Japan) according to the manufacturer's protocol.

For the evaluation of serum exosomes of OS patients and healthy donors on the migration and invasion of 143B and U_2_OS, cells (6 × 10^4^) were respectively seeded into the non-coated upper chamber of transwell plates (8 mm pore size; Corning) for a migration assay and into matrigel coated upper chamber (BD Bioscience, 354,234) for an invasion assay with 50 μg/ml exosomes. For the evaluation of exosomes derived from OS on the migration and invasion of 143B and U_2_OS, the same procedure as above mentioned was performed with 10 μg/ml and 50 μg/ml. After culturing for 24 h, cells were fixed with methanol and stained with a 0.1% crystal violet solution. Migrated cells were evaluated in five fields per well under a microscope.

### Wound-healing assay

Confluent 143B cell monolayers were scratched with a sterile 100-µl pipette tip and treated with or without GW4869 at a dose of 1 μg /ml. Cell migration was monitored for 24 and 48 h under a microscope. The widths of the ‘wound’ (scratched areas) were measured and the proportion of wound healing was calculated by the following formula: 100%-(width after 24 h/width at the beginning) × 100%.

### ELISA analysis of Sr-exosomal PD-L1 expression

**Fig. 1:**
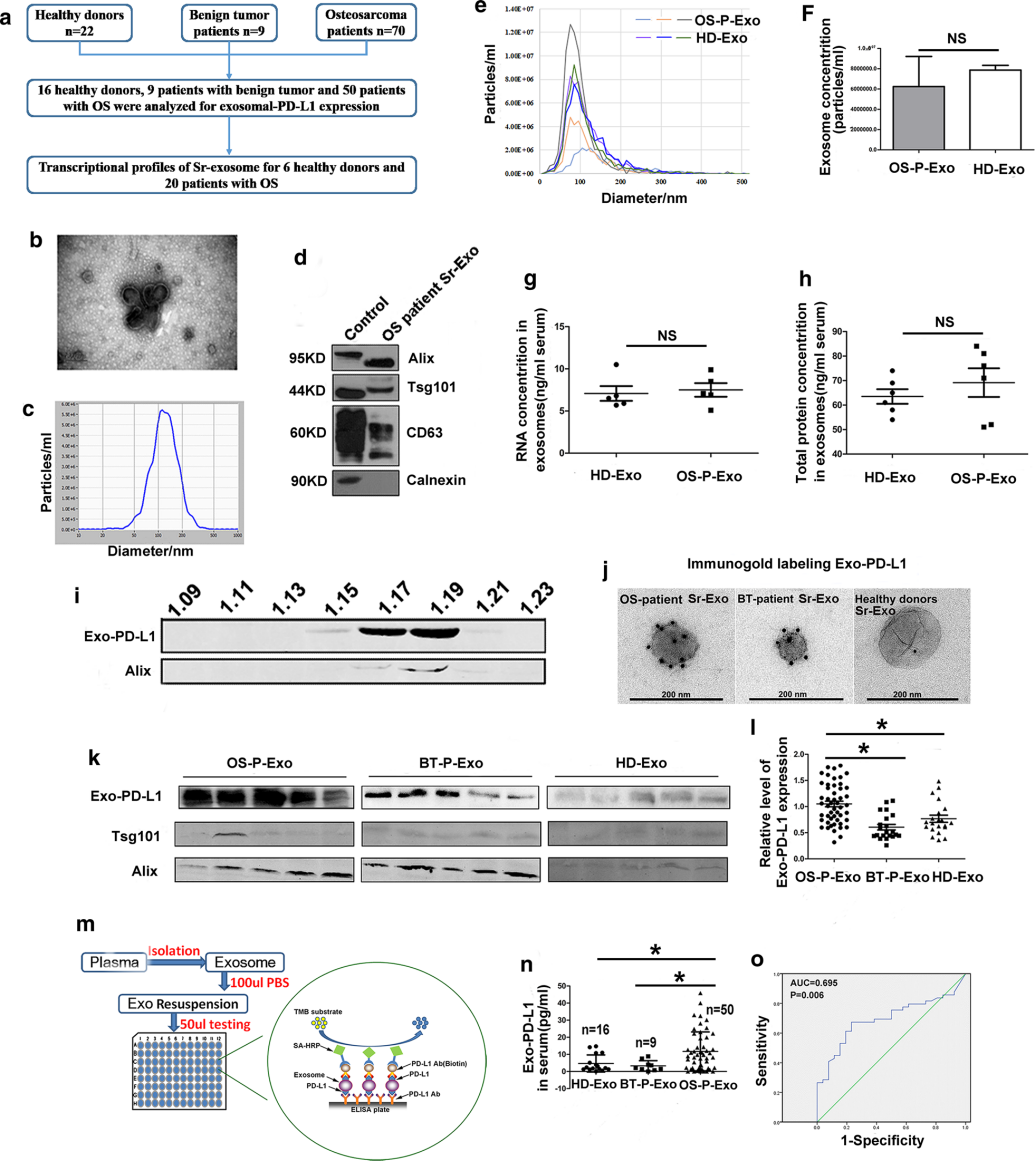
Higher level of Sr-exosomal PD-L1 in OS patients compared to healthy donors and patients with benign tumor. **a** Plan for analysis of patients with OS and benign tumor and healthy donors; **b** Electron microscopy showing vesicles of 30–100 nm diameter as exosomes; **c** Nanoparticle tracking system for evaluation of exosome concentration and size; **d** Western blot analysis for the representative biomarkers of exosomes; **e, f** Analysis of particle concentration of Sr-exosomes between OS patients and healthy donors by nanoparticle tracking system; G-H. Concentration of RNA and total protein in Sr-exosomes between OS patients and healthy donors; **I** Density gradient centrifugation confirming that PD-L1 existing in Sr-exosomes derived from OS patients; **j** A representative TEM image of Sr-exosomes immunogold-labelled with PD-L1 antibody derived from healthy donors and patients with osteosarcoma and benign tumor; **k**, **l** Western blot analysis showing Sr-exosomal PD-L1 expression of healthy donors and patients with OS and benign tumors; **m** Schematic of ELISA to measure PD-L1 concentration in serum exosomes derived from healthy donors and patients with osteosarcoma and benign tumor; **n** Levels of PD-L1 in serum exosomes derived from healthy donors and patients with osteosarcoma and benign tumors by ELISA; **o** ROC analysis indicating Sr-exosomal PD-L1 having an AUC of 0.695 to distinguish OS patients from healthy donors

For detection of PD-L1 in exosomes, Sr-exosomal PD-L1 from OS patients and healthy donors was analyzed by ELISA. Exosomes were isolated as above mentioned method and then resuspended in 100 µl PBS. Fifty µl suspension was analyzed as the schematic diagram shown in Fig. 1m. We equilibrated all materials and prepared reagents to room temperature prior to use. All standards, controls and samples were analyzed in duplicate. The protocol was followed as the instruction (ab214565, Human PD-L1 SimpleStep ELISA^®^ Kit). Calculate the average absorbance value for the blank control (zero) standards. Subtract the average blank control standard absorbance value from all other absorbance values. Create a standard curve by plotting the average blank control subtracted absorbance value for each standard concentration (y-axis) against the target protein concentration (x-axis) of the standard. Use graphing software to draw the best smooth curve through these points to construct the standard curve. Determine the concentration of the target protein in the sample by interpolating the blank control subtracted absorbance values against the standard curve. Multiply the resulting value by the appropriate sample dilution factor, if used, to obtain the concentration of target protein in the sample. Highest standard should be further diluted and reanalyzed. Similarly, samples which measure at an absorbance values less than that of the lowest standard should be retested in a less dilute form.

### Pulmonary metastatic model study

All protocols were approved by the animal care committee of the Peking University Health Science Center. We have minimized the number of animals used and their suffering.

Experiment NO 1 in vivo, in order to evaluate the effect of 143B-derived exosomes and an inhibitor of exosome secretion GW4869 on pulmonary metastasis, 143B cells were injected into tail vein of 6-week-old female BALB/c nude mice (Vitalriver, Beijing, China) in a volume of 10 µL (50,000 cells) as the primary injection. Administration of PBS, GW4869 (at a dose of 2.5 mg/kg, from day 3 to day 21, once three days, seven times in total. Sigma-Aldrich, cat. D1692) and 143B-derived exosomes (at a dose of 10 μg per mouse, from day 3 to day 21, once three days, seven times in total) as secondary injection was performed after primary injection. Mice were euthanized at four weeks after the secondary injection. The procedure was shown in Fig. 6b.

Experiment NO 2 in vivo, the model and treatment were followed as the flowchart indicated in Fig. 8a. The mice were sacrificed at four weeks after secondary injection. The lung specimen were stored at − 80 ℃ for experiments.

### Haematoxylin and eosin staining

Mice were sacrificed at four weeks (Experiment NO 1 and 2 in vivo) after secondary injection and the lung tissue was dissected. The tissue was fixed in 10% formalin at room temperature overnight and consequentially dehydrated in 70% ethanol. Samples were mounted in paraffin blocks and sectioned at 5 mm of thickness. Slides were baked at 60 ℃ for 15 min, then treated with Xylene Substitute (Citrisolv) (Fisher chemical cat. x3p-1GAL) then rehydrated and stained with Hematoxylin, followed with washes with running water. An incubation with Acid Alcohol (Sigma, cat. A3429) was performed, followed with washes with running water. The samples were then stained with eosin (Thermo Scientific) then dehydrated with ethanol followed by Xylene Substitute (Citrisolv). Samples were mounted with Permount (Fisher Scientific, cat. SP15-500). Slides were submitted for pathologic evaluation. Pathologist was blinded to the sample.

The mouse weight was recorded for analysis and lungs were collected for H&E staining. Pulmonary metastasis was evaluated by visually counting the number of metastatic nodules, maximum diameter per metastatic nodule and cross-section area per metastatic nodule in the entire mouse lung section for each mouse, using Nikon NIS-Elements software (Nikon Corporation Instruments, Tokyo, Japan).

### Statistical analysis

Statistical analysis was conducted using SPSS 19.0 (SPSS Inc., USA). All data presented as mean ± SD unless described otherwise. Two sided Student t test or one-way ANOVA was performed using SPSS 19.0. Receiver operating characteristic curve (ROC) analysis was used to assess the ability of exosomal-PD-L1 to discriminate OS patients from healthy donors and exosomal-PD-L1, exosomal N-cadherin and exosomal N-cadherin/E-cadherin to discriminate OS patients with pulmonary metastasis from ones without metastasis. Statistical significance was defined as *p* < 0.05.

## Results

### Confirming characteristics of serum exosomes (Sr-Exo) of OS patients and OS cell line exosomes

The plan for analysis of patients with OS, benign tumor and healthy donors (HD) was shown in the flowchart (Fig. 1a). The exosome fraction was characterized by transmission electron microscopy (TEM) and nanoparticle tracking system for morphology, concentration and size, as well as by western blot analysis for exosome associated biomarkers. As nanoparticle tracking analysis was shown in Figs. 1c, 5b, the size distribution of these Sr-Exo of OS patients and OS cell line-derived exosomes were mostly around 100 nm. TEM showed that the extracellular vesicle fraction from OS patient serum and osteosarcoma cells, contained typical cup-shaped vesicles, which were exosomes (Figs. 1b, 5c). Exosomes from 143B and U_2_OS cell lines and OS patient serum were positive for the representative biomarkers (i.e. Tsg101, Alix, CD63 and CD9) in western blot analysis that were among the top 100 most frequently detected exosomal proteins (shown at website, www.exocarta.org) (Fig. 1d, 5d). In summary, the above mentioned experiments confirmed the extracellular vesicle extracted from cell line media and clinical serum samples as the exosomes.

### Confirmation of PD-L1 existence in exosomes isolated from OS patient serum and osteosarcoma cell lines

Although PD-L1 is well known to be upregulated in many tumor tissues, such as melanoma, breast cancer and lung carcinama, few studies have been focused on osteosarcoma, especially circulatory PD-L1 delivered by exosomes. We firstly confirmed the existence of PD-L1 in exosomes isolated from OS patient serum and osteosarcoma cell lines. The analysis of density gradient centrifugation experiment confirmed that PD-L1 existed in exosomes derived from OS patients (Fig. 1i). By immunogold labeling for Sr-exosomal PD-L1, we detected PD-L1 in exosomes derived from healthy donors and patients with OS and benign tumor, whereas PD-L1 positive exosomes from healthy donors and benign tumor patients were fewer than that from OS patients (Fig. 1j).

Western blot analysis was performed for four osteosarcoma cell lines and the result indicated that HOS, KHOS, 143B and U_2_OS cell lines showed stable PD-L1 protein expression (Fig. 5f). Meanwhile, the immunogold labeling for exosomal PD-L1 confirmed PD-L1 existence in four osteosarcoma cell lines (Fig. 5e). All these experiments confirmed PD-L1 existed in the exosomes derived from OS patient serum and osteosarcoma cell lines.

### Higher level of Sr-exosomal PD-L1 in OS patients compared to healthy donors and patients with benign tumor

In order to reveal whether several parameters of exosomes can distinguish OS patients from healthy donors, we performed the following analysis. We compared the particle concentration of exosomes between OS patients and healthy donors and the result showed there was no significant difference between them by using the nanoparticle tracking system analysis (Fig. 1e, f). We also found there was no difference in total protein or mRNA level in these exosomes (Fig. 1g, h).

Next, we evaluated whether Sr-exosomal PD-L1 from OS patients was higher than that of healthy donors and benign tumor patients. Western blot analysis showed Sr-exosomal PD-L1 expression of OS patients was significantly higher than that of healthy donors and benign tumor patients (*p* < 0.05; Fig. 1k, l). Schematic of ELISA to measure PD-L1 concentration in exosomes isolated from healthy donors and patients with benign tumor and OS was shown in Fig. 1m. We also found that the level of Sr-exosomal PD-L1 from 50 OS patients was significantly higher than that of healthy donors and benign tumor patients in ELISA analysis (*p* < 0.05; Fig. 1n). Furthermore, receiver operating characteristic curve (ROC) indicated that Sr-exosomal PD-L1 showed an AUC (Area Under Curve) of 0.695 (95% CI 0.577–0.814) to distinguish OS patients from healthy donors (*p* = 0.006, Fig. 1o). The result of ROC indicated a strong correlation between Sr-exosomal PD-L1 level and osteosarcoma in ELISA analysis. Thus, these results encouraged us to perform further analysis to reveal the potential mechanism of Sr-exosomes from OS, especially PD-L1 contained in Sr-exosomes, for OS pathogenesis and pulmonary metastasis.

### Higher level of Sr-exosomal PD-L1 in osteosarcoma pulmonary metastasis patients compared to ones without metastasis

**Fig. 2:**
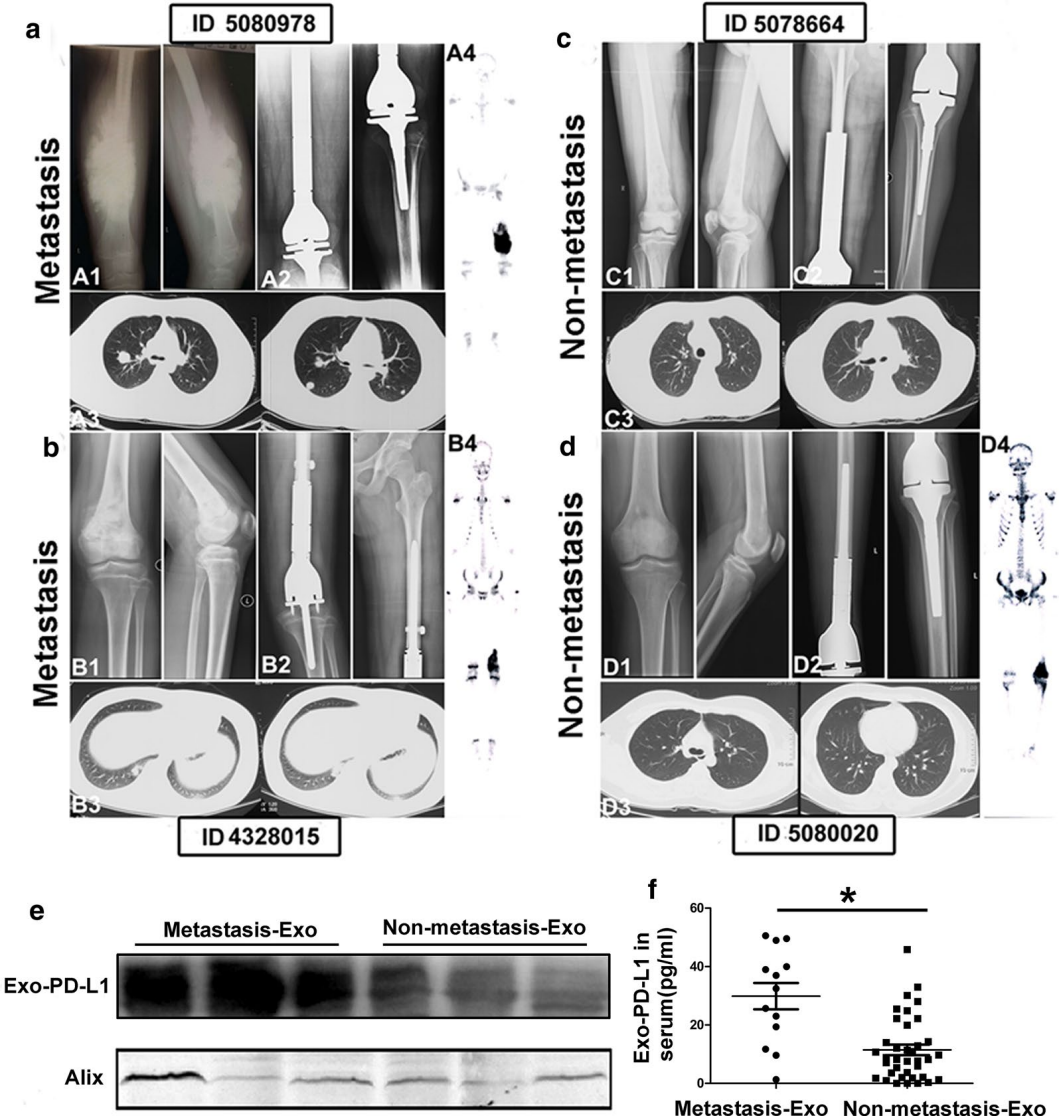
Higher level of Sr-exosomal PD-L1 in OS patients with pulmonary metastasis compared to ones without metastasis. **a**–**d** Radiological images of four OS patients with and without pulmonary metastasis. **e** Western blot analysis revealing significant difference for Sr-exosomal PD-L1 level in serum exosomes derived from patients with and without pulmonary metastasis; **f** ELISA analysis showing higher level of exosomal PD-L1 in serum of pulmonary metastasis patients compared to ones without metastasis

In light of the highly distinguishing OS patients from healthy donors and benign tumor patients, we next evaluated whether level of Sr-exosomal PD-L1 expression could identify patients with pulmonary metastasis from ones without metastasis at diagnosis. The radiological images of four OS patients with and without pulmonary metastasis were shown in Fig. 2a–d. Western blot and ELISA analysis confirmed the existence of PD-L1 in exosomes from OS patient serum and there was significant difference for the level of exosomes derived from patients with metastasis compared to those without metastasis (*p* < 0.05, Fig. 2e, f).

### Bioinformatic analysis of global mRNA expression of Sr-exosome in osteosarcoma patients and healthy donors

**Fig. 3:**
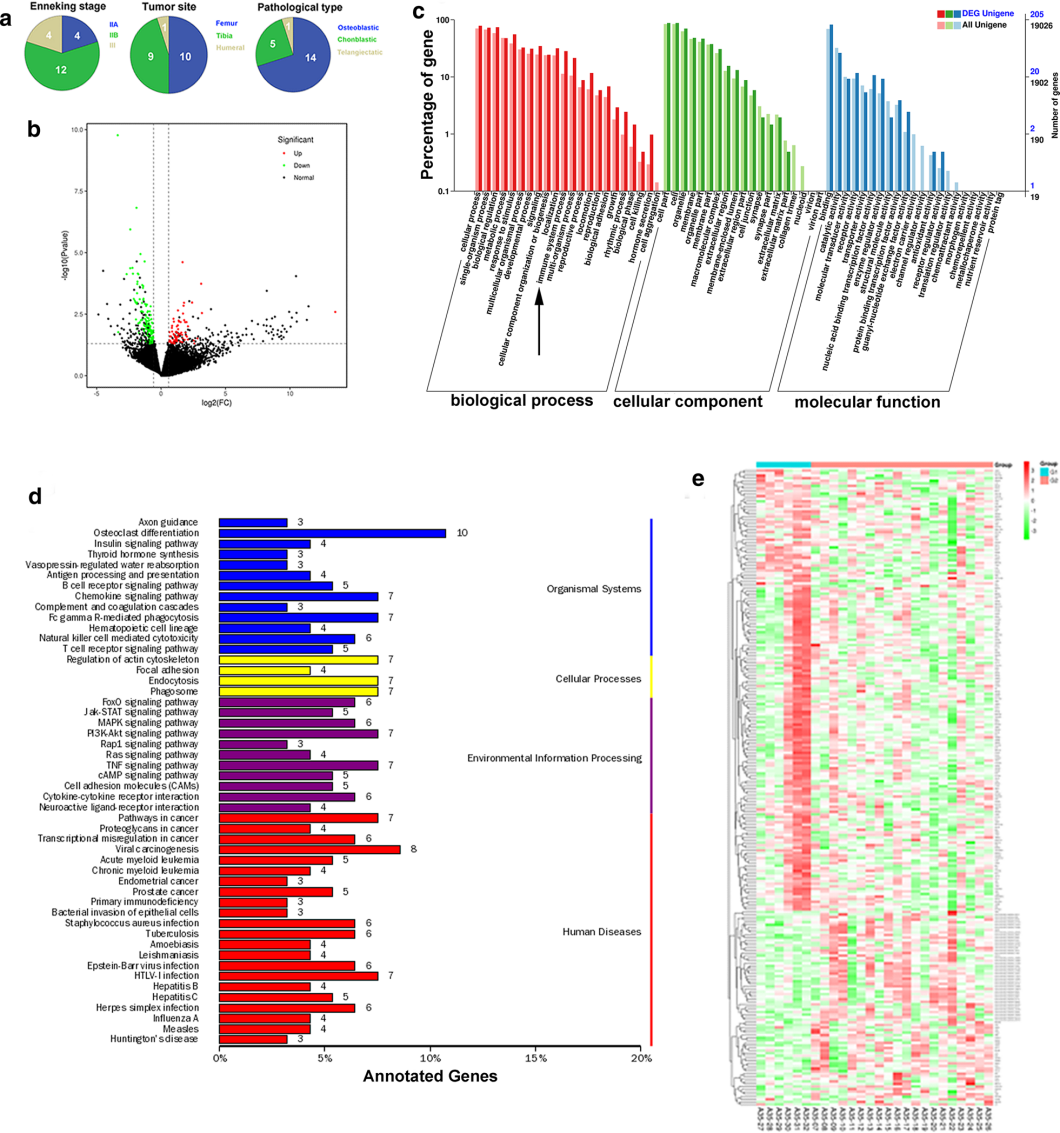
Bioinformatic analysis of global mRNA expression in Sr-exosomes of twenty osteosarcoma patients and six healthy donors. **a** Clinical characteristics of OS patients, which received Sr-exosome RNA sequence; **b** Volcano plots of differently expressed genes in Sr-exosomes of six healthy donors and twenty OS patients; **c** GO analysis demonstrating Sr-exosomes of OS patients involving in the process of immune function, which was indicated by arrow; **d** KEGG pathways analysis revealing the enrichment in Sr-exosomal mRNA between OS patients and healthy donors; **e** Hierarchical clustered heatmap of differently expressed genes in Sr-exosomes of six healthy donors and twenty OS patients

This study was designed to perform the global mRNA screening, which included exosomes isolated from serum of twenty OS patients and six healthy donors. The clinical characteristics of twenty OS patients were shown in Fig. 3a. Our research provided a new insight into RNA sequence in exosomes and may make a little contribution to the understanding of exosomal mRNA composition in OS patients. We found the differential expression of RNAs in exosomes, significant KEGG pathways and GO enrichment between OS patients and healthy donors. Bioinformatic analysis revealed 248 mRNAs as exclusively significant expression in the Sr-exosomes derived from OS patients compared to ones from healthy donors (Additional file 1: Table-S1). Volcano plots showed the differently expressed genes in the Sr-exosomes bewteen six healthy donors and twenty OS patients. Meanwhile, log2 fold change > 1 with statistical significance (*p* < 0.05) was detected by RNA-seq using NGS. Significantly upregulated and downregulated genes were respectively colored in red and green. X axis: log2 fold change of gene expression. Y axis: statistical significance of the differential expression in the scale of − log10 (*p* value) (Fig. 3b).

GO analysis demonstrated that Sr-exosomes of OS patients involved in the process of immune function during osteosarcoma pathogenesis, which was pointed by arrow shown in Fig. 3c. All above mentioned bioinformatics results illustrated Sr-exosomes from OS patients may be involved in osteosarcoma pathogenesis, especially the immune process in the tumor microenviroment. The analysis of KEGG pathways revealed that which was enriched in mRNA and the number of according mRNAs were labeled for each pathway. It revealed that several mRNAs in Sr-exosomes derived from OS patients were enriched in the cancer-associated and immune-associated pathways (Fig. 3d). Heatmap of differential mRNA expression showed 75 Sr-exosomal mRNAs were identified to be highly expressed in OS patients compared to healthy donors, while 173 Sr-exosomal mRNAs were low expression in OS patients compared to healthy donors (Fig. 3e).

### Establishment of co-expression network centered with PD-L1 among Sr-exosomal differently expressed mRNA between twenty osteosarcoma patients and six healthy donors

**Fig. 4:**
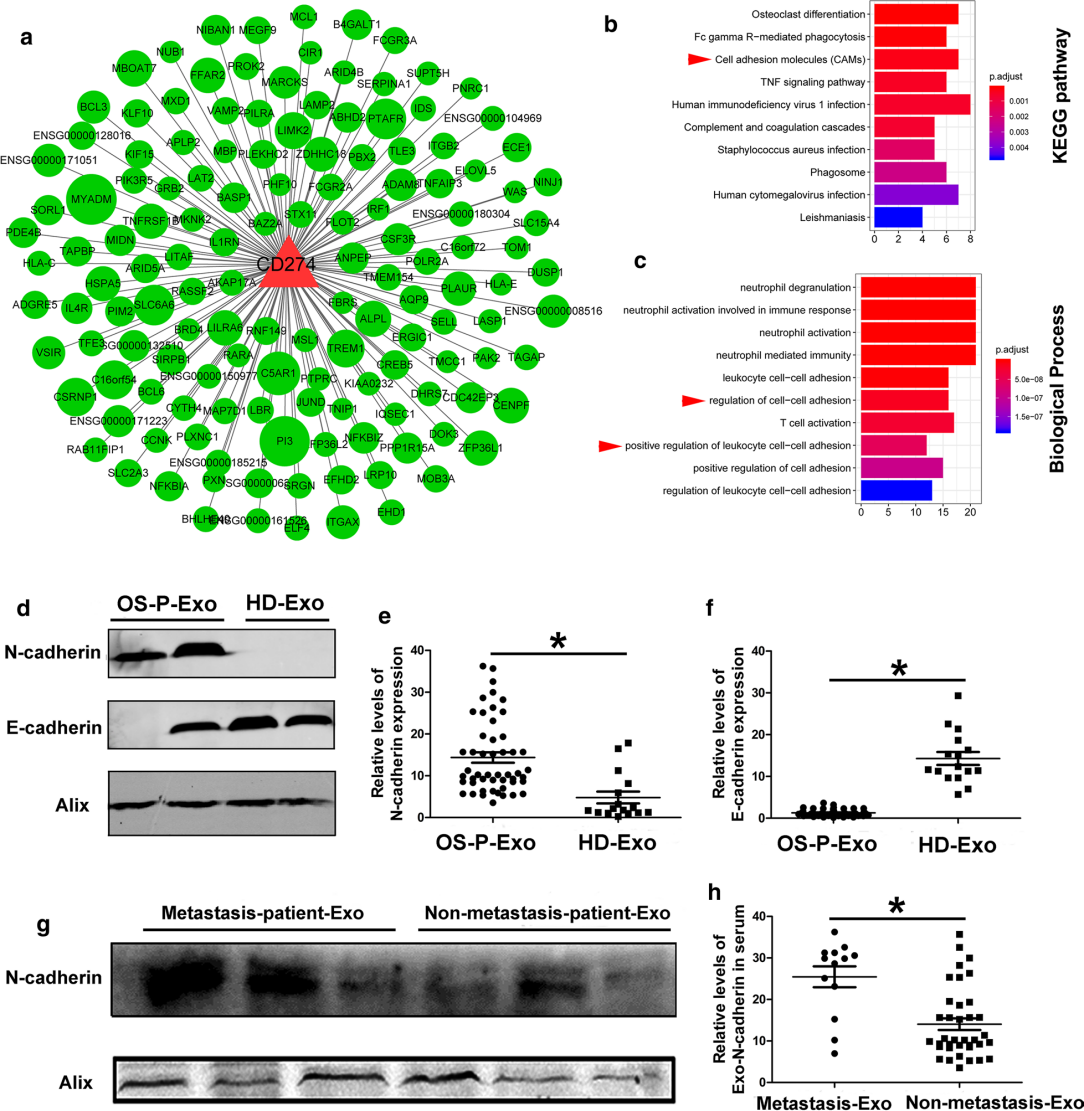
Establishment of co-expression network centered with PD-L1 among Sr-exosomal differently expressed mRNA between twenty OS patients and six healthy donors. **a** Co-expression network centered with PD-L1 among the differently expressed genes; **b** KEGG analysis showing the cell adhesion co-expression with PD-L1, which was indicated by red arrow head; **c** Biological process including regulation of cell–cell adhesion and positive regulation of leukocyte cell–cell adhesion, which was also indicated by red arrow head; **d**–**f** Higher expression of exosomal N-cadherin and lower expression of exosomal E-cadherin in OS patients compared to healthy donors; **g**, **h**. Higher expression of exosomal N-cadherin in OS patients with metastasis compared to ones without metastasis

Based on the bioinformatic DEG data of Sr-exosomes between twenty OS patients and six healthy donors, we established the co-expression network among the differently expressed genes, which had PD-L1 as the core gene (Fig. 4a). Because the higher level of exosomal PD-L1 was observed in OS patients and OS pulmonary metastasis patients, we centered PD-L1 as the core gene in the co-expression network. From the co-expression network, we found some genes to be closed to core gene PD-L1 and it illustrated that Sr-exosomal PD-L1 may play an important role in osteosarcoma pathogenesis. KEGG analysis showed the function of cell adhesion may be co-expressed with PD-L1 in the progression of osteosarcoma, which was indicated by red arrow head (Fig. 4b) and biological process also included regulation of cell–cell adhesion and positive regulation of leukocyte cell–cell adhension, which was also shown by red arrow head (Fig. 4c). These bioinformatic results demonstrated that exosomal PD-L1 had the closed relationship with the cell–cell adhesion function in the pathogenesis of osteosarcoma.

### Confirmation of existence of N-cadherin in exosomes derived from osteosarcoma patient serum

On the basis of results of co-expression network centered by exosomal PD-L1, it illustrated that cell–cell adhesion function had the closed relationship with PD-L1 in the osteosarcoma-derived exosomes. Thus, we detected the expression of exosomal N-cadherin and E-cadherin in OS patient serum. The level of exosomal N-cadherin expression in OS patients was significantly higher than that in healthy donors (*p* < 0.05, Fig. 4 d, e). The opposite trend of exosomal E-cadherin expression was observed in OS patients (*p* < 0.05, Fig. 4d, f). Furthermore, for OS patients with pulmonary metastasis, the level of exosomal N-cadherin expression was also obviously higher than that of patients without pulmonary metastasis (*p* < 0.05, Fig. 4g, h). These results demonstrated Sr-exosomal N-cadherin had the same trend with Sr-exosomal PD-L1 in exosomes derived from OS patients.

### Exosomes derived from osteosarcoma cell line and patient serum induce osteosarcoma migration and invasion and increase N-cadherin and PD-L1 expression in OS cells

**Fig. 5:**
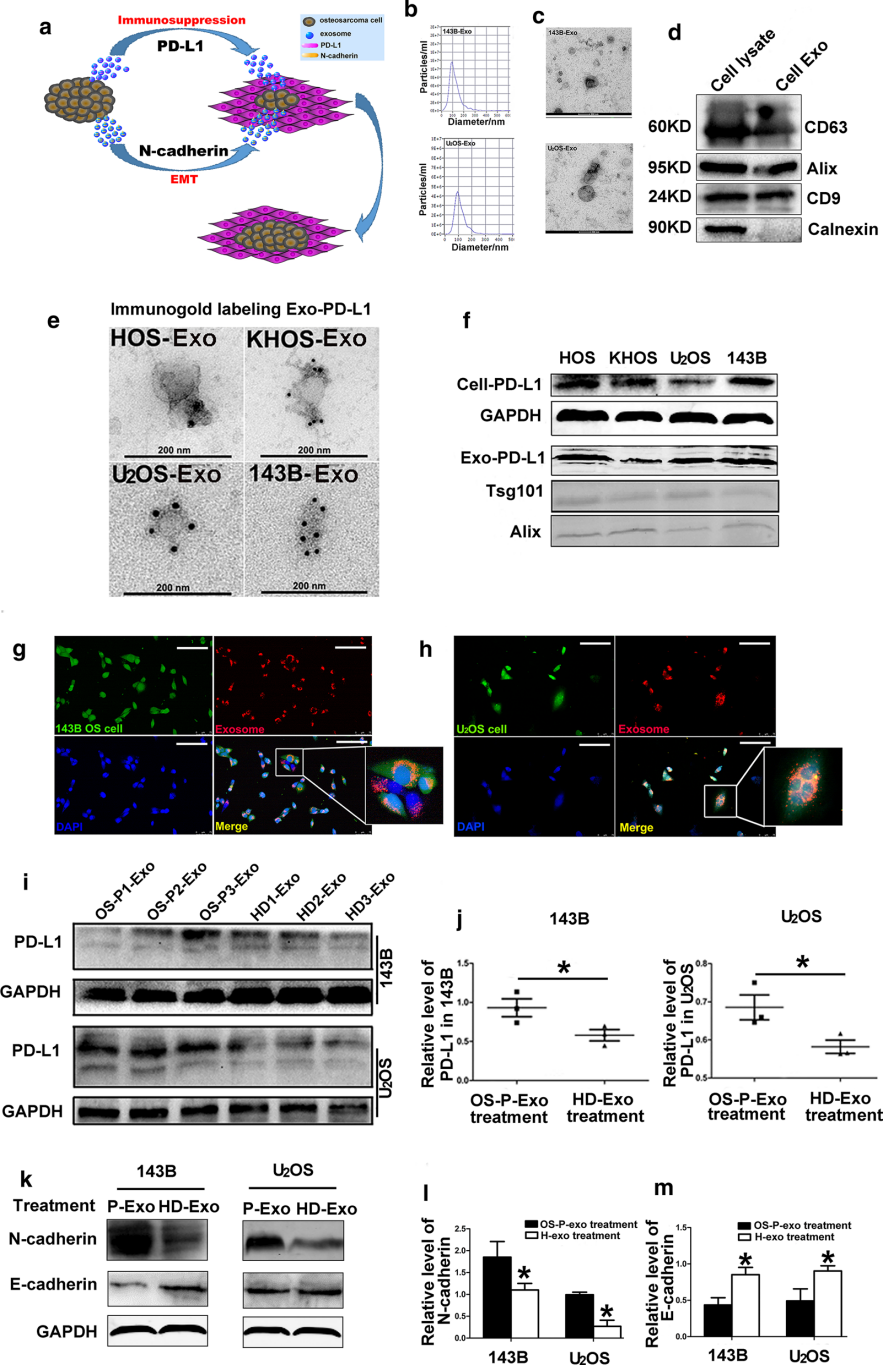
Effect of exosomes from OS patient serum on the cellular PD-L1, N-cadherin and E-cadherin expression in 143B and U_2_OS. **a** Schematic diagram showing the effect of osteosarcoma-derived exosomes on the metastatic lesions; **b** Nanoparticle tracking system for concentration and size of exosomes derived from medium of 143B and U_2_OS; **c** Electron microscopy showing exosomes isolated from medium of 143B and U_2_OS; **d** Western blot analysis for the representative biomarkers of exosomes; **e** The representative TEM image of exosomes immunogold-labelled with anti-PD-L1 antibodies isolated from HOS, KHOS, 143B and U_2_OS; **f** Western blot analysis showing PD-L1 expression in four osteosarcoma cell lines and their exosomes; **g**, **h** Immunofluorescent analysis detecting internalization of exosomes from 143B cells or OS patient serum to the recipient 143B and U_2_OS cells; **i, j** Influence of Sr-exosome isolated from OS patients on PD-L1 expression in 143B and U_2_OS cells; **k**–**m** Influence of Sr-exosome isolated from OS patients on N-cadherin and E-cadherin expression in 143B and U_2_OS cells

As mechanism of epithelial mesenchymal transformation (EMT) and effect of PD-L1 immunosuppression play a pivotal role in tumor metastasis, we hypothesize osteosarcoma stimulates the metastatic process by releasing exosomes which carry large amount of PD-L1 and N-cadherin to help the progression and migration of metastatic tumors (Fig. 5a).

We first tested if exosomes derived from OS cell line and patient serum were internalized to the recipient 143B and U_2_OS cells. 143B and U_2_OS cells respectively labeled with CFSE (green) were treated with red PKH26-labeled exosomes isolated from 143B cell line and patient serum. Immunofluorescent microscopy analysis revealed the presence of PKH26 labeled exosomes from 143B cells or patient serum exclusively in the cytoplasm of 143B and U_2_OS cells, especially around the perinuclear region (Fig. 5g, h). Thus, we concluded that the recipient cells were inundated with the exogenous exosomes. These results indicated that osteosarcoma cells can internalize the exogenous exosomes.

Cell viability was examined for different concentrations of Sr-exosomes derived from the OS patient and healthy donor to treat 143B and U_2_OS cells for 24, 48 and 72 h. Sr-exosomes derived from OS-P (steosarcoma patient) did not affect the viability at low and high doses of exosomes compared to ones derived from HD (healthy donor) (*p* > 0.05, Additional file 2: Figure S1). Next, we determined the effect of exosomes isolated from the OS patient/healthy donor serum and OS cell line on the migration and invasion of 143B and U_2_OS. Firstly, we detected the effect of exosomes isolated from serum of three OS patients on osteosarcoma cell lines. The purified Sr-Exo from OS patients significantly increased cell migration and invasion compared to Sr-Exo of healthy donors (*p* < 0.05, Additional file 3: Fig. S2a, b). Then likewise, the purified exosomes from 143B cell media statistically induced cell migration and invasion in a dose-dependent manner for 143B and U_2_OS cells compared to control (exosome-depleted media group) (*p* < 0.05, Additional file 4: Fig. S2c, d).

Next, we used exosomes isolated from serum of OS-P and HD to treat 143B and U_2_OS in order to reveal the change of cellular PD-L1, N-cadherin and E-cadherin. We found cellular PD-L1 and N-cadherin in 143B and U_2_OS were obviously upregulated after treatment of Sr-exosomes from OS-P compared to that from HD (*p* < 0.05, Fig. 5i–l). Meanwhile, cellular E-cadherin of 143B and U_2_OS after OS-P-Exo treatment was not increased compared to HD-Exo treatment (*p* < 0.05, Fig. 5k, m). These results illustrated the migration and invasion abilities of osteosarcoma could be increased after osteosarcoma-derived exosomes treatment.

### Osteosarcoma-derived exosomes promote pulmonary metastasis and a pharmacological inhibitor of exosome secretion GW4869 decreases osteosarcoma metastasis in metastatic mouse models

**Fig. 6:**
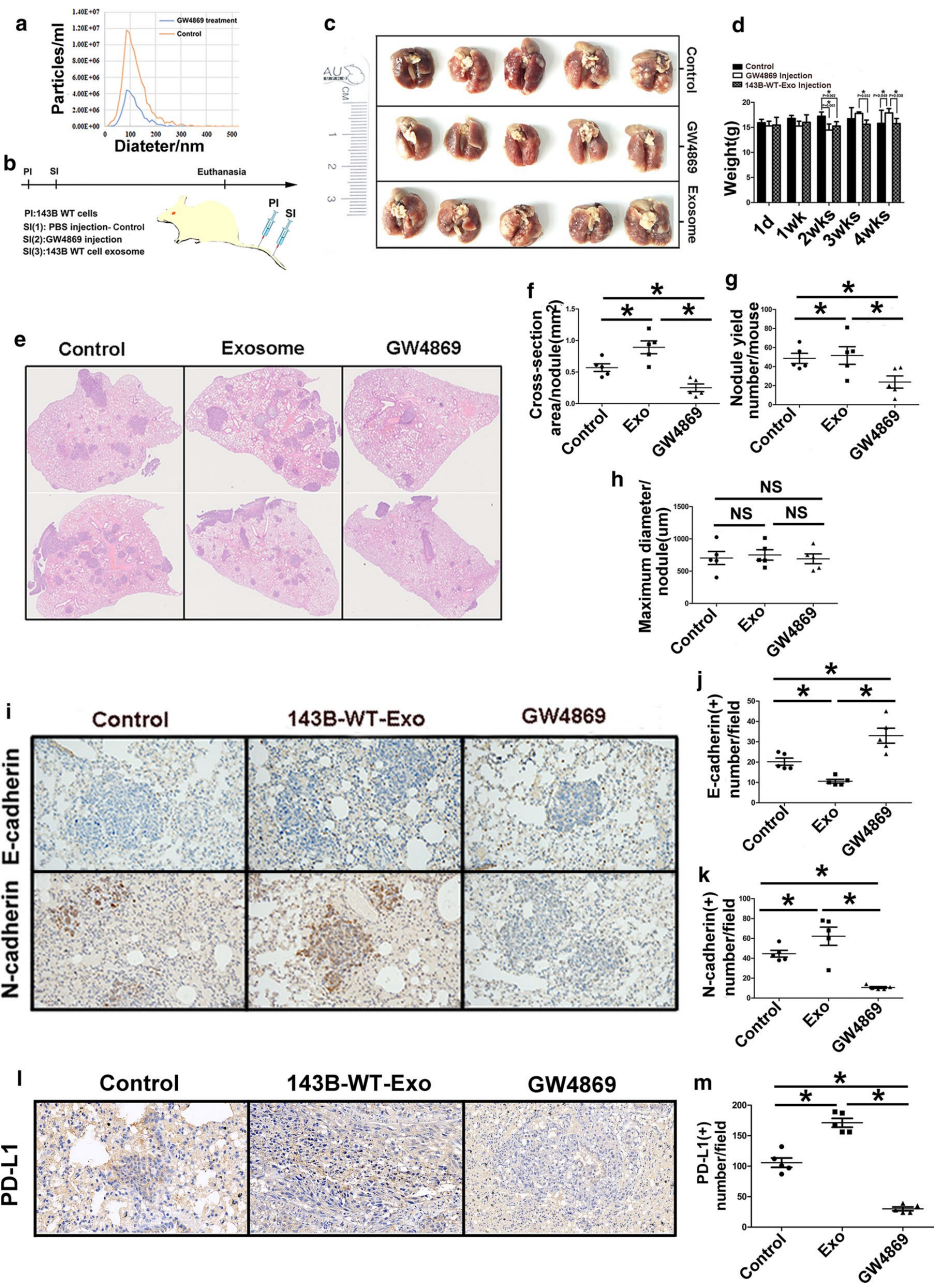
Effect of exosome secretion inhibitor GW4869 on the progression of osteosarcoma pulmonary metastasis. **a** Nanoparticle tracking analysis showing inhibition of 143B exosome secretion by utilizating GW4869; **b** The flowchart showing the plan to test GW4869 on pulmonary metastasis nodules and metastatic lesion area in vivo; **c** Gross specimens of lungs in three groups; **d** Weight change of mice in three groups; **e** Haematoxylin and eosin staining to show the metastatic lesions in the lung of three groups; **f**–**h** Maximum diameter, number and areas of metastatic lesion nodules in three groups; **i**–**k** Upregulation of E-cadherin and downregulation of N-cadherin were observed in the metastatic lesion treated by GW4869; **l**, **m** Suppression of PD-L1 expression at the pulmonary metastatic lesions by administration of GW4869 and upregulation by the treatment of exosomes from 143B cells. Experiments were performed using 5 mice for each group. Data represent mean ± SD

To reveal the effect of GW4869, which was a small molecule inhibitor of exosome secretion, on the migration of 143B cells in vitro, it showed that GW4869 could not influence the migration ability of 143B cells (Additional file 4: Fig S3a–c). Whereas nanoparticle tracking analysis revealed that the concentration of exosomes produced by 143B cells which was treated with GW4869 was lower than that of control group, it illustrated GW4869 suppressed osteosarcoma cells releasing exosomes (Fig. 6a). Next, we determined the effect of exosomes derived from osteosarcoma cells and GW4869 on OS metastasis in the metastatic model, in which the treatment procedure was shown in the flowchart (Fig. 6b). Gross specimens of lungs in three groups were shown in Fig. 6c and we could observe more metastatic nodules at the surface of lung in control and 143B-Exo treated groups compared to ones in GW4869 treated group. Mice weight in GW4869 treated group was significantly higher than that of control and 143B-Exo treated groups at three and four weeks after secondary injection (Fig. 6d). Treatment of 143B tumor-bearing mice with GW4869 significantly reduced pulmonary metastatic nodule number and metastatic lesion area in vivo compared to control group (*p* < 0.05). Meanwhile, injection with 143B-Exo could increase metastatic lesion nodule number and area compared to control and GW4869 treated groups (*p* < 0.05, Fig. 6e–g). However, we found no significant difference of maximum diameter among GW4869 treated, 143B-Exo treated and control groups (Fig. 6h). Furthermore, the upregulation of E-cadherin and downregulation of N-cadherin was observed in the metastatic lesion treated by GW4869 and the inverse condition occurred in 143B-Exo treated group (*p* < 0.05, Fig. 6i–k). The PD-L1 expression of pulmonary metastatic lesions in the model can be suppressed by the administration of GW4869 and upregulated by the treatment of 143B-Exo (*p* < 0.05, Fig. 6l–m). These results illustrated that exosomes derived from OS could stimulate osteosarcoma cells to disseminate to the lung and GW4869 can be used to inhibit the secretion of exosomes from osteosarcoma cells in the tumor microenviroment. Thus, it may be a new targeted therapy to reduce the pulmonary metastasis in the future.

### Influence of knockdown of Rab27a and PD-L1 on 143B cells

**Fig. 7:**
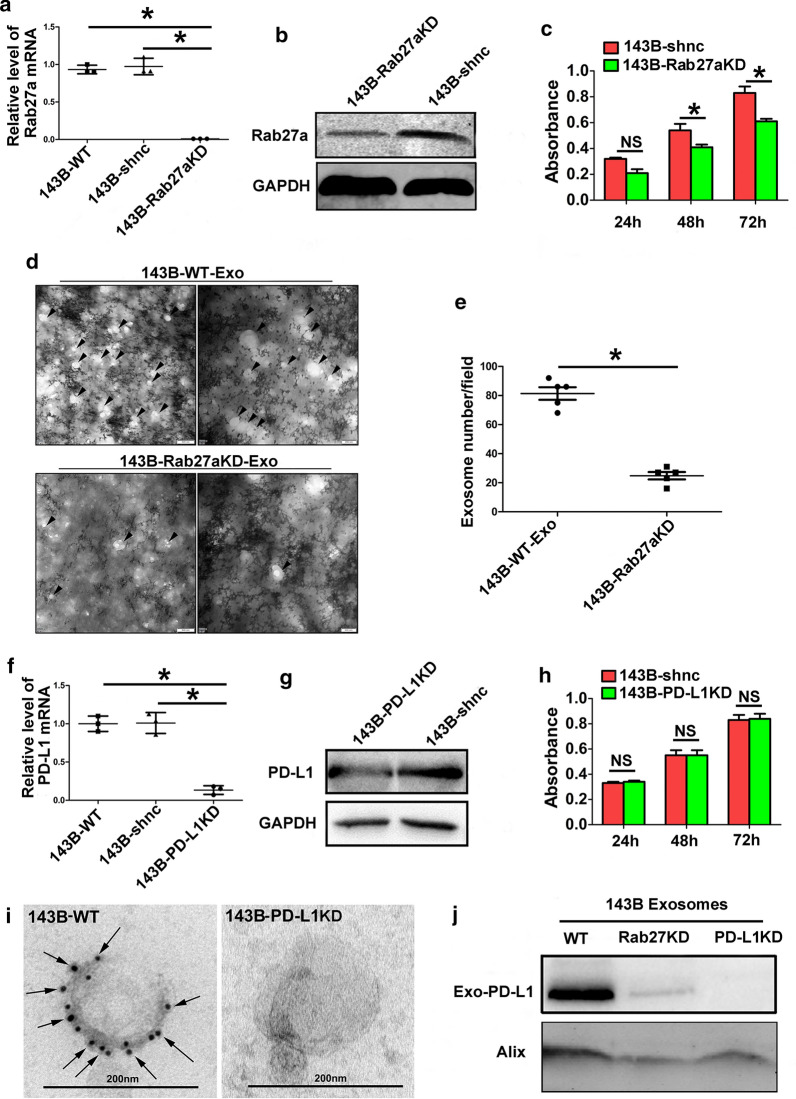
Influence of knockdown of Rab27a and PD-L1 on 143B cell. **a**, **b** Confirmation of knockdown of 143B Rab27a (143B-Rab27aKD) by CRISPER-Cas9 by qPCR and western blot analysis; **c **Cell Counting Kit 8 assay showing 143B-Rab27aKD proliferating slower than 143B-shnc; **d, e**. Representative TEM images showing there was fewer exosomes in the supernate isolated from 143B-Rab27aKD; **f, g**. Confirmation of knockdown of 143B PD-L1 (143B-PD-L1KD) by CRISPER-Cas9 by qPCR and western blot analysis; **h** knockdown of 143B PD-L1 exerting no influence on the proliferation; **i** Comparision of exosomal PD-L1 expression isolated from 143B-PD-L1KD and 143B-WT by immunogold labeling; **j** Exosomal PD-L1 expression for three osteosarcoma cell lines, including 143B-WT, 143B-Rab27aKD and 143B-PD-L1KD

It is well recognized that Rab27a is an important protein which could regulate the exosome release. Knockdown of 143B Rab27a (143B-Rab27aKD) by CRISPER-Cas9 was confirmed by qPCR and western blot analysis (Fig. 7a, b). TEM analysis showed there was fewer exosomes in the supernate of 143B-Rab27aKD and it also confirmed the knockdown validity of regenerated cell line of 143B-Rab27aKD (Fig. 7d, e). Furthermore, 143B-Rab27aKD proliferated slowly than 143B-shnc in CCK-8 assay and knockdown of Rab27a possibly influenced not only the exosome secretion, but also proliferation of 143B cells (Fig. 7c. Meanwhile, knockdown of 143B PD-L1 (143B-PD-L1KD) by CRISPER-Cas9 was also confirmed by qPCR and western blot analysis (Fig. 7f, g). We found less PD-L1 existed on the surface of exosomes isolated from 143B-PD-L1KD compared to 143B-WT by immunogold labeling for PD-L1 (Fig. 7i). Otherwise, knockdown of PD-L1 would not affect the proliferation of osteosarcoma cells (Fig. 7h). Importantly, we revealed that knockdown of Rab27a and PD-L1 could blocked exosomal PD-L1 secretion (Fig. 7j). These results confirmed the knockdown validity of regenerated cell lines of 143B-Rab27aKD and 143B-PD-L1KD and they can be used in the pulmonary metastasis models in the next experiments.

### Osteosarcoma-derived exosomes and exosomal-PD-L1 facilitate pulmonary metastasis in metastatic models

**Fig. 8:**
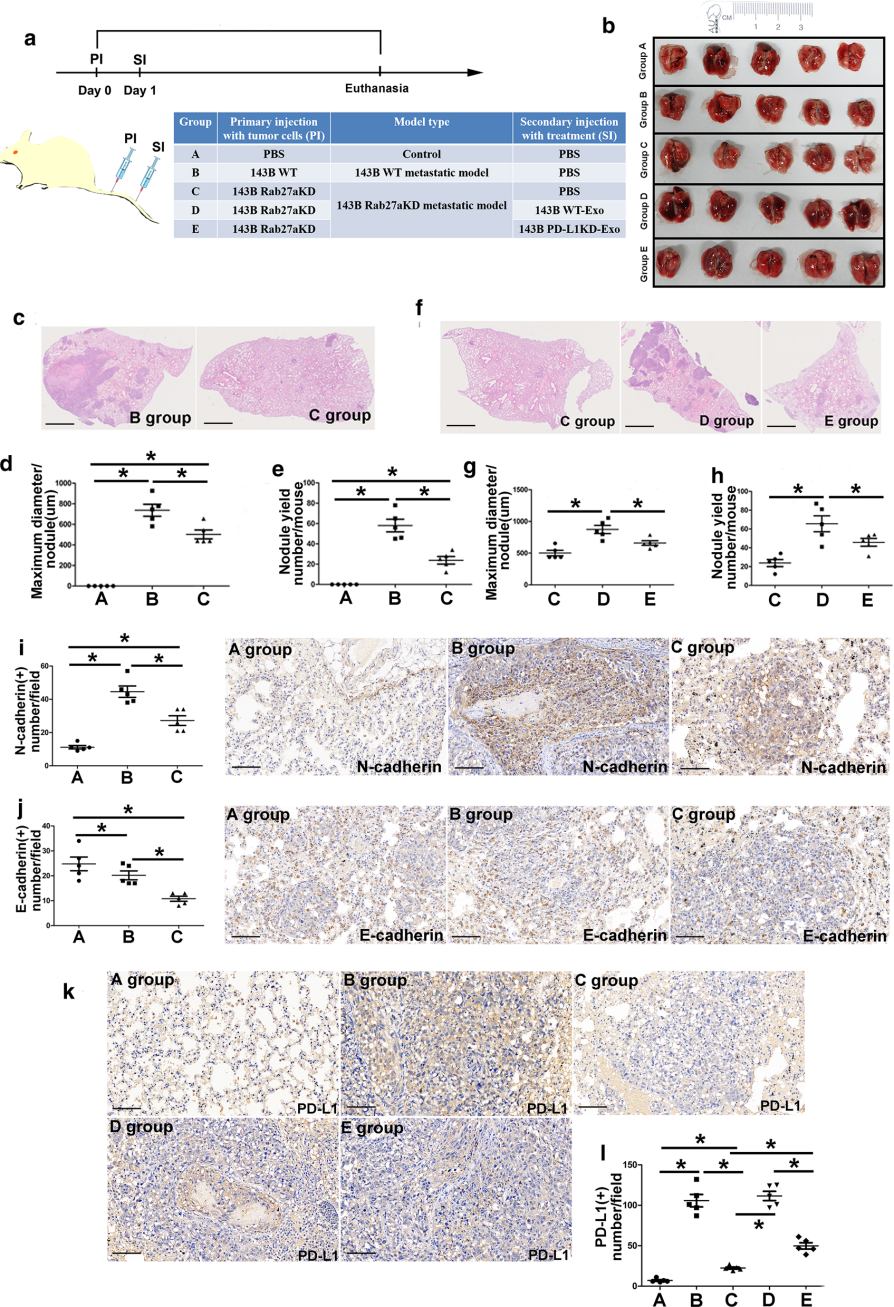
Osteosarcoma-derived exosomes and exosomal-PD-L1 facilitate pulmonary metastasis in metastatic models A. **a** flowchart showing plan to investigate the effect of osteosarcoma-derived exosomes and exosomal PD-L1 on pulmonary metastasis in vivo; **b**. Gross specimens of lungs in five groups; **c**. Representative HE images showing metastasis of 143B-Rab27aKD in BALB/C mice after injection of PBS, exosomes derived from 143B-WT and 143B-Rab27a in Groups A, B and C; **d, e**. Maximum diameter of metastatic lesion and number of metastasis nodules in three groups; **f** Effect of exosomes derived from 143B-PD-L1KD in 143B-Rab27aKD model; **g–h** Maximum diameter of metastatic lesion and number of pulmonary metastasis nodules in C, D and E groups. **i**, **j**. Expression of N-cadherin and E-cadherin at the metastatic lesion in Group A, B and C; **k, l** Influence of exosomes and exosomal PD-L1 on PD-L1 expression of metastatic lesions. Experiments were performed using 5 mice for each group. Data represent mean ± SD

To investigate the effect of OS-derived exosomes on the pulmonary metastasis in vivo, we performed different 143B metastastic models in nude mice as the flowchart and they received respective treatments (Fig. 8a). Importantly, knockdown of Rab27a influenced the release of exosomes and further blocked exosomes and exosomal PD-L1 secretion (Fig. 7j). We injected PBS, 143B-WT and 143B-Rab27aKD into tail vein as the primary injection to acquire Control, 143B-WT and 143B-Rab27aKD metastastic models. Then, injection of PBS, 143B-WT-Exo and 143B-PD-L1KD-Exo as the secondary injection revealed the effect of different exosomes on the pulmonary metastasis (Fig. 8a).

By utilization of metastastic models, we can evaluate the effect of OS-derived exosomes on the pulmonary metastasis among A, B and C groups. Likewise, the assessment of the influence of exosomal PD-L1 on the lung metastasis can be achieved among C, D and E groups. Secretion of OS exosomes decreased in Group C due to knockdown of Rab27a. Gross specimens of lungs were shown in Fig. 8b. Then we found the number of pulmonary metastatic nodules and maximum diameter of metastatic lesion in Group C were significantly depressed compared to Group B (*p* < 0.05, Fig. 8c–e). Furthermore, the formation of pulmonary metastatic nodules in Group E, for which injection of 143B-PD-L1KD-Exo was performed, was obviously inhibited compared to that in Group D which was receiving 143B-PD-L1WT-Exo. It illustrated that exosomal PD-L1 could stimulate the pulmonary metastasis in osteosarcoma pulmonary metastasis models. Moreover, injection of 143B-WT-Exo and 143B-PD-L1KD-Exo in Group D and E can induce the progression of pulmonary metastasis compared to Group C and it demonstrated that the OS-derived exosomes and exosomal PD-L1 can facilitate the progression of pulmonary metastasis (Fig. 8f–h).

The expression of N-cadherin at the metastatic lesion in Group B was significantly higher than that in Group C and E-cadherin had the opposite trend (Fig. 8i–k). It illustrated knockdown of Rab27a could suppress exosome release and decrease N-cadherin expression at the metastatic lesion. Next, administration of 143B-WT-Exo in Group D could supply exosomal PD-L1 at the metastatic lesion of 143B-Rab27aKD model compared to that in Group E receiving 143B-PD-L1KD-Exo (Fig. 8l, m). These results showed that exosomal PD-L1 could increase the expression of PD-L1 at the metastatic lesions due to the exogenous package of PD-L1 in the osteosarcoma-derived exosomes.

### Change of exosomal PD-L1 for osteosarcoma patients between pre-treatment and post-treatment

As the exosomal PD-L1 and N-cadherin had the important effect in the progression of pulmonary metastasis, we were aiming to demonstrate the clinical diagnostic value for treatment and prediction of pulmonary metastasis.

**Fig. 9:**
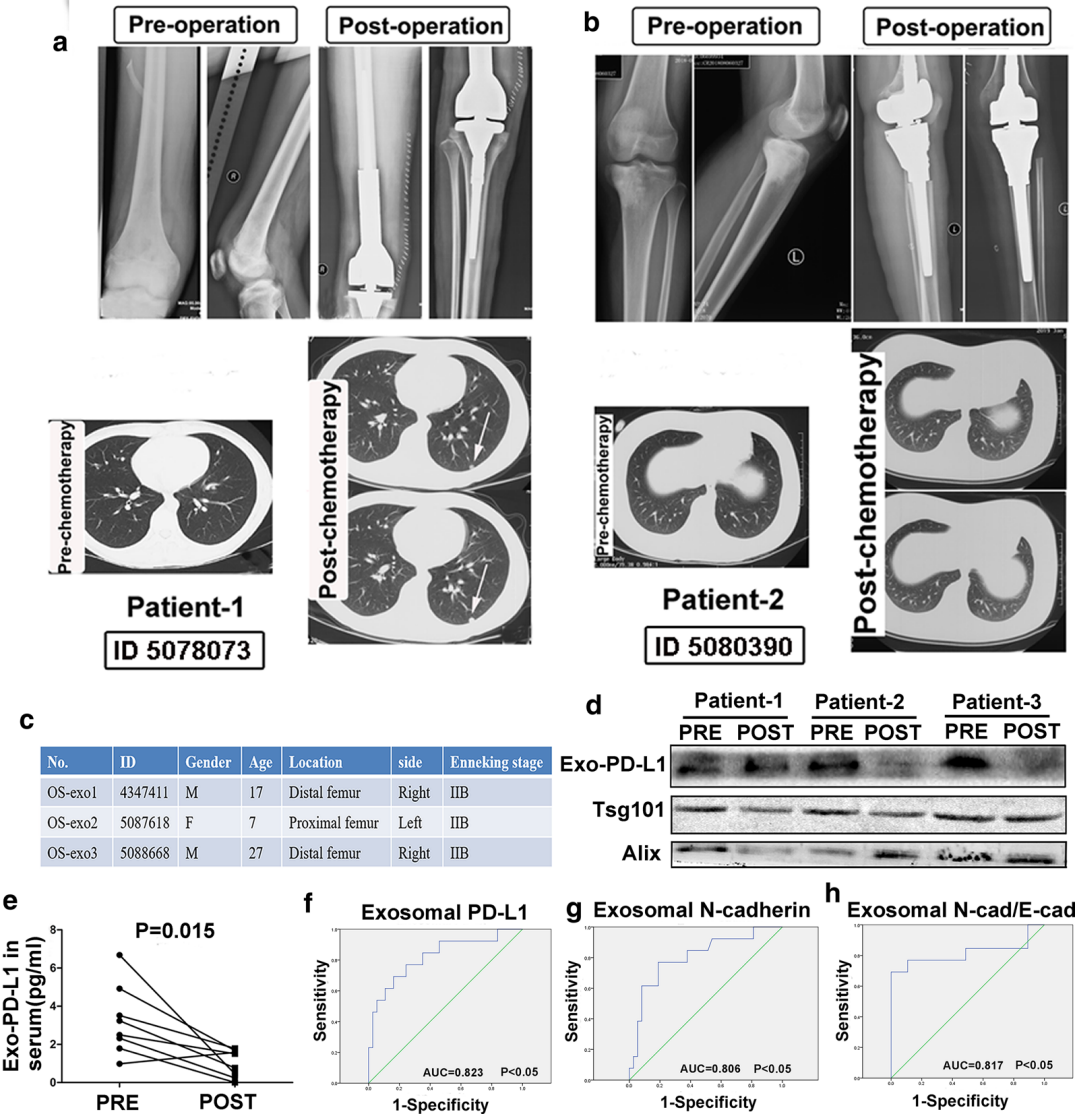
Change of exosomal PD-L1 for OS patients between pre-treatment and post-treatment. **a, b** Follow-up for patient 1 with disease progression and patient 2 with stable disease; **c** Clinical information of three typical OS patients; **d** Sr-exosomal PD-L1 expression by western blot analysis for patients 1, 2 and 3 during the follow-up. **e **Level of Sr-exosomal PD-L1 expression before and after chemotherapy; F–H. ROC analysis of exosomal PD-L1, exosomal N-cadherin and exosomal N-cadherin/E-cadherin

The change of exosomal PD-L1 expression during time course was next evaluated between pre-treatment and post-treatment. We found that the level of exosomal PD-L1 was significantly downregulated after neoadjuvant chemotherapy and surgical treatment (*p* < 0.05, Fig. 9e). The clinical information of three typical cases was shown in Fig. 9c. Western blot analysis also revealed exosomal PD-L1 expression was significantly lower after neoadjuvant chemotherapy and surgical treatment, whereas some patients would not show obvious decrease of PD-L1 expression (eg, Patient 1). The disease was stable in the lung assessment for Patient 2 and 3 during the follow-up (Fig. 9b) and the level of exosomal PD-L1 after treatment was obviously lower than that of pre-treatment (eg, Patient 2 & 3, Fig. 9d). The radiological images detected the pulmonary metastasis after neo-chemotherapy for Patient 1 (Fig. 9a). These findings illustrated that the change of exosomal PD-L1 expression in OS patients was related to the disease progression during the follow-up.

Receiver-operating characteristic curve (ROC) analysis of exosomal PD-L1 and N-cadherin was performed to distinguish OS patients with pulmonary metastasis (n = 13) from ones without metastasis (n = 37). ROC analysis showed AUC of 0.823 for exosomal PD-L1 (Fig. 9f), 0.806 for exosomal N-cadherin (Fig. 9g) and 0.817 for exosomal N-cadherin/E-cadherin (Fig. 9h) to distinguish OS patients with pulmonary metastasis from ones without metastasis. Furthermore, the statistical analysis showed that the level of exosomal PD-L1, N-cadherin and ratio of exosomal N-cadherin/E-cadherin could significantly distinguish OS patients with pulmonary metastasis from ones without metastasis (*p* < 0.05, shown in Fig. 9f–h).

**Fig. 10:**
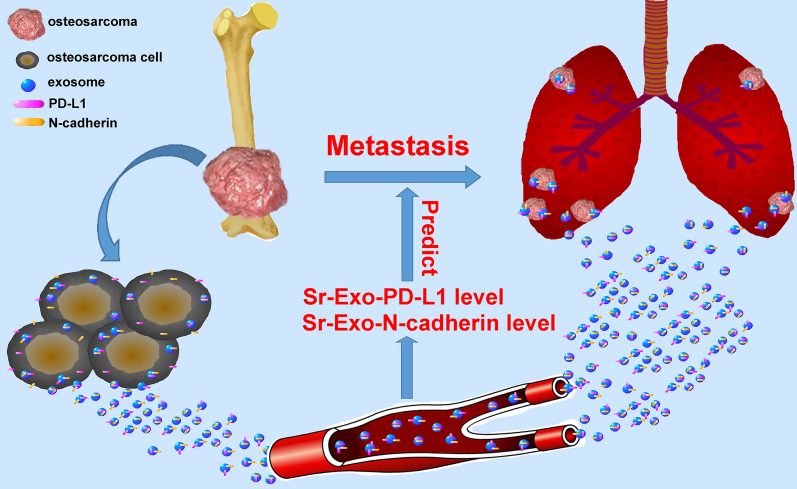
Schematic diagram showed the outline of osteosarcoma pulmonary metastasis process in which osteosarcoma-derived exosomes involved. Osteosarcoma released its exosomes into the microenvironment and osteosarcoma-derived exosomes carried PD-L1 and N-cadherin to the lung through the circulatory system. Osteosarcoma cells at the metastatic lesions of lung internalized exosomes transmitted from the primary osteosarcoma and these exosomes assisted the migration and progression of metastatic tumors. Detection of exosomal PD-L1 and N-cadherin levels in the circulatory system could probably predict the occurrence of osteosarcoma pulmonary metastasis

Schematic diagram showed the outline of osteosarcoma pulmonary metastasis process in which osteosarcoma-derived exosomes involved (Fig. 10). The present study was demonstrating one important mechanism of osteosarcoma pulmonary metastasis by releasing exosomes which packaged cellular PD-L1 and N-cadherin into the extracellular vesicle. Osteosarcoma released its exosomes into the microenvironment and osteosarcoma-derived exosomes carried PD-L1 and N-cadherin to the lung through the circulatory system. Osteosarcoma cells at the metastatic lesions of lung internalized exosomes transmitted from the primary osteosarcoma and these exosomes assisted the migration and progression of metastatic tumors. Detection of exosomal PD-L1 and N-cadherin levels in the circulatory system could probably predict the occurrence of osteosarcoma pulmonary metastasis.

## Discussion

Osteosarcoma commonly occurs in children and young adults and pulmonary metastasis occurs in approximately 15–20% of patients [28]. Clinical outcomes of osteosarcoma treatment have not significantly improved over twenty years. It is necessary to detect the effective marker to predict the prognosis for OS patients. To date, no individual molecular marker has been demonstrated to have the prognostic significance than the current clinical markers. In the literature, several recent studies have identified that exosomal miRNAs, mRNAs and preteins are strikingly stable and have a potential ability to predict the prognosis of patients with malignant tumors [6, 17, 29].

It has been reported that osteosarcomas may communicate with their environment by generating exosomes [30–34]. Studies of tumor-derived exosomes may possibly reveal newly important targets for cancer therapy, as well as perhaps yield tumor antigens that are diagnostic or prognostic for tumor aggressiveness [25, 26]. However, specific isolation of exosomes from serum of cancer patients remains a challenge due to lack of specific markers that can be used to identify and distinguish cancer exosomes from ones produced by normal cells. In the literature, Melo et al. reported a cell surface proteoglycan, glypican-1 (GPC1) which was specifically enriched in cancer cell-derived exosomes, to detect the early stage of pancreas cancer with high sensitivity and specificity [35]. However, lack of good biomarker of the sarcoma lead us to detect a better approach to screen the prognosis of OS patients. Notably, our results provided clear visual evidence of PD-L1 existing in the exosomes produced by osteosarcoma cells using electron transmission microscopy of immunogold labeling. Likewise, western blot analysis also showed that these exosomes expressed PD-L1. Furthermore, we can use Sr-exosomal PD-L1 to distinguish OS patients from patients with benign tumors and healthy people, with ROC analysis revealing an AUC of 0.695. This finding illustrates that Sr-exosomal PD-L1 can be used as the important biomarker to distinguish OS patients from healthy people and has the potential ability to predict the progression of osteosarcoma.

Several studies revealed the presence of PD-L1 in exosomes isolated from serum of cancer patients and the relationship between PD-L1 level and pathological features [24, 25, 36, 37]. Chen et al. reported metastatic melanomas released more exosomes, which delivered PD-L1 on their surface. Furthermore, stimulation with interferon-γ increased the amount of PD-L1 on these vesicles, which could suppress the function of CD8^+^ T cells and facilitated tumor growth. It also showed that the level of circulating exosomal PD-L1 positively correlated with IFN-γ level in the serum of patients with metastatic melanoma and varied during the course of anti-PD-1 therapy. The number of tumor-infiltrating CD8^+^ T lymphocytes decreased significantly after injection of exosomes. Troyer et al. reported the negative effect of OS exosomes on CD4^+^ and CD8^+^ T cell proliferation was much greater than that of osteoblast derived exosomes. These studies suggested that exosomal PD-L1 systemically suppressed anti-tumor immunity function and stimulated the cancer progression [25, 38]. In the present study, we showed in the pulmonary metastasis model, mice injected with 143B-WT cells had abundant metastatic lesions in the lung compared to ones receiving the injection of 143B-Rab27aKD cells, which failed to secrete more exosomes. It also illustrated that exosomes derived from osteosarcoma facilitated the progression of pulmonary metastasis. Afterwards, we found that injection of exosomes derived from 143B-PD-L1KD cells was obviously inhibiting the pulmonary metastasis compared to that receiving the injection of exosomes from 143B-WT cells in the metastatic model. These results showed that the exosomal PD-L1 can stimulate the pulmonary metastasis in the osteosarcoma pulmonary metastasis model.

Personalized medicine strategies are widely advocated for the treatment of OS patients with pulmonary matastasis [39–43]. However, we are currently faced with rigorous problem due to lack of excellent therapy which can control the tumor development [44–47]. Detecting a good personalized medicine strategy is promising for osteosarcoma patients. Liu et al. reported A485, a p300/CBP inhibitor abrogated immune process and decreased the secretion of exosomal PD-L1 by blocking the transcription of CD274, which combined with the anti-PD-L1 antibody to reactivate T cells function for tumor attack [26]. Thus, suppression of exosomal PD-L1 may be a promising personalized therapy. It has been reported that GW4869 could decrease the secretion of tumor cell exosomes and inhibit the cell growth [48]. Although we can not observe the significantly inhibiting effect of GW4869 on the osteosarcoma cell growth in vitro in the present study, inhibition of the exosome secretion by GW4869 could suppress the pulmonary metastasis in vivo study. It may be related to the effect of inhibiting osteosarcoma exosome secretion in the microenviroment for OS patients.

In the present study, bioinformatic analysis demonstrated that Sr-exosomes isolated from OS patients may involve in the important process of immune function and cancer pathogenesis. Furthermore, it showed 248 mRNAs differently expressed in Sr-exosomes derived from OS patients compared to that from healthy donors and several mRNAs were enriched in cancer and immune associated pathways. These results illustrated that Sr-exosomal mRNAs derived from OS patients may participate in the osteosarcoma pathogenesis, especially the immune process in the microenviroment. Interestingly, PD-L1 in Sr-exosomes of OS patients was significantly higher than that of patients with benign tumors and healthy donors. This result confirmed exosomes and exosomal PD-L1 may be related to the pathogenesis of osteosarcoma from the clinical perspective.

Afterwards, based on the bioinformatic mRNA DEG (differently expressed gene) data of serum exosomes between twenty OS patients and six healthy donors, we established the co-expression network among the differently expressed genes, which had PD-L1 as the core gene. From the co-expression network, we found the function of cell–cell adhesion was associated with exosomal PD-L1. It is well established that the complex process of osteosarcoma metastasis involves multiple steps during which osteosarcoma cells spread from primary tumor to other organs. Epithelial–mesenchymal transition (EMT) process, which is also referred to as “mesenchymalization”, is an important metastasis mechanism of osteosarcoma by which osteosarcoma cells could stimulate the epithelial cell transition from adherent epithelial to mobile mesenchymal states and this mechanism facilitates cancer cells dissemination at the metastatic lesions. The pathological process of EMT involves a series of molecular and genetic changes in tumor cells that induce a conversion of the epithelial phenotype to a highly aggressive and invasive mesenchymal state at the metastatic lesions. The molecular change includes the expression levels of cell surface proteins, such as a loss of E-cadherin and the appearance of N-cadherin. Notably, cadherin switch from E-cadherin to N-cadherin is a key step occurring during EMT process and increase the pulmonary metastasis progression. Gain of function of N cadherin was considered as a universal hallmark of EMT process and it was reported to promote tumorigenesis of different cancers. Although it is well known that N cadherin function may prove important in multiple cancers, the exact regulation of exosomal N cadherin in osteosarcoma remains largely unknown. In the present study, we found the expression of exosomal N-cadherin in OS patients was significantly higher than that in healthy donors. Furthermore, for OS patients with pulmonary metastasis, their expression of exosomal N-cadherin was also obviously higher than that of patients without pulmonary metastasis. As N-cadherin plays a pivotal role in tumor metastasis, we hypothesize osteosarcoma stimulates the metastatic process by releasing exosomes which carry large amount of N-cadherin to help the progression and migration of metastatic tumors. Thus, we used exosomes derived from OS patient serum to treat 143B and U_2_OS cells. We found exosomes isolated from OS patient serum and 143B cells could induce the migration and invasion of osteosarcoma in vitro. The result also showed that the expression of N-cadherin in 143B and U_2_OS cells after OS-P-Exo treatment was significantly higher than that after HD-Exo. Otherwise, the expression of E-cadherin in 143B and U_2_OS cells had the opposite trend. In the metastatic models, we found the level of N-cadherin expression at the metastatic lesion of 143B-WT model was significantly higher than that of 143B-Rab27aKD model. Rab27a is reported to be a critical gene for organelle-specific protein trafficking in extracellular vesicle [49]. Knockdown of Rab27a in osteosarcoma cells could decrease the secretion of osteosarcoma exosomes and 143B-Rab27aKD cells lacks the ability of releasing of exosomal N-cadherin and PD-L1. This change may suppress the progression of metastatic lesions, which was also confirmed in the present study. Notably, in our hypothesis, when the pulmonary metastasis occurs, osteosarcoma cells metastalizing to lung is first step and the second step is the metastatic osteosarcoma cells in the lung internalization of exosomes transmitted from the primary osteosarcoma. The first step of osteosarcoma cells metastalizing to lung is prerequisite. If there was no osteosarcoma cells metastalizing to lung, osteosarcoma cell derived exosomes could not launch and stimulate the process of pulmonary metastasis.

In this study, we demonstrated that PD-L1 and N-cadherin co-existed in exosomes from serum of OS patients. Meanwhile, exosomal PD-L1 and N-cadherin exerted an important effect which induced the pulmonary metastasis. In the clinical practice, we lack an accurate biomarker to predict the occurrence of pulmonary metastasis for OS patients during the follow-up. It has been reported that several miRNAs, mRNAs and proteins isolated from osteosarcoma exosomes could predict the progression of pulmonary metastasis [17, 50]. We analyzed exosomal PD-L1, exosomal N-cadherin and ratio of exosomal N-cadherin/E-cadherin by receiver-operating characteristic curve analysis to distinguish patients with metastasis from ones without metastasis. The result revealed that diagnostic effectiveness of exosomal PD-L1 was the highest one among the three parameters, followed by ratio of exosomal N-cadherin/E-cadherin and exosomal N-cadherin. This could contribute more to help us to predict the occurrence of pulmonary metastasis for OS patients. In the clinical practice, we commonly face with a condition that we need more parameters to confirm one problem in order to increase the diagnostic sensitivity and specificity. Due to immature exosome testing in the clinical practice, we want to provide more related biomarkers to predict the osteosarcoma progression. Thus, we develop biomarkers of exosomal PD-L1, exosomal N-cadherin and exosomal N-cadherin/E-cadherin and increase the diagnostic sensitivity and specificity for disease progression of OS patients.

There were several limitations in the present study. It has been reported that the immunosuppression and EMT are the important mechanisms of tumor metastatic progression [13, 51, 52]. However, in the present study it did not evaluated how exosomes from osteosarcoma interacted with the immune cells. The study of interaction of exosomes derived from osteosarcoma with immune cells is processing in our team. Furthermore, we found there was significant difference of Sr-exosomal PD-L1 level in patients with metastasis compared to those without metastasis. However, the case number is a little smaller that we need to collect more samples to reveal the more objective results for assessment of exosomal PD-L1 and bioinformatics analysis. To date, we are following up all twenty OS patients who received the sequence of mRNA in exosomes and want to find several significant genes which can predict the patient survival and reveal more information about the relationship between different expressed genes and follow-up information of osteosarcoma patients. It can also further test whether exosomal PD-L1 could have a good relationship with patient survival. Lastly, in the further study, we need more cases to test the diagnostic effectiveness of exosomal PD-L1, ratio of exosomal N-cadherin/E-cadherin and exosomal N-cadherin.

## Conclusion

In summary, osteosarcoma stimulates pulmonary metastasis by releasing exosomes, that carry PD-L1 and N-cadherin. Detection of exosomal PD-L1 and N-cadherin from serum of OS patients may predict pulmonary metastasis progression for OS patients.

## Supplementary information

**Additional file 1: Table S1**. OS patient Sr-exosome vs healthy donor Sr-exosome DEG mRNA.

**Additional file 2: Figure-1.** Treatment of 143B and U2OS cells for 24, 48 and 72 hours using different concentration of Sr-exosomes derived from OS patient and healthy donor.

**Additional file 3: Figure-2 A-B**. Effect of exosomes isolated from serum of OS patient and healthy donor on the migration and invision of 143B and U2OS in vitro; C-D. Effect of different concentration of exosomes isolated from 143B cell on the migration and invision of 143B and U2OS in vitro.

**Additional file 4: Figure-3 A-B**. Effect of the small molecule of exosome secretion inhibitor GW4869, on the wound healing of 143B cells in vitro, indicating no influence of GW4869 on the wound healing; C. no influence of GW4869 on the migration of 143B cells in vitro.

## Data Availability

The datasets used and/or analyzed during the current study are available within the manuscript and its supplementary information files.

## Abbreviations

OS: Osteosarcoma
Sr-exosome or Sr-Exo: Exosomes derived from serum
PD-L1: PROGRAMMED death-ligand 1
TEM: Transmission electron microscopy
EV: Extracellular vesicle
HD: Healthy donor
OS-P: Osteosarcoma patient
Exo: Exosome
ROC: Receiver operating characteristic curve
AUC: Area under curve
