# Supplementary figures and images for: Exosomal PD-L1 and N-cadherin predict pulmonary metastasis progression for osteosarcoma patients
Source: J Nanobiotechnology. 2020 Oct 22;18:151. doi: 10.1186/s12951-020-00710-6 (PMC7579953; doi:10.1186/s12951-020-00710-6)

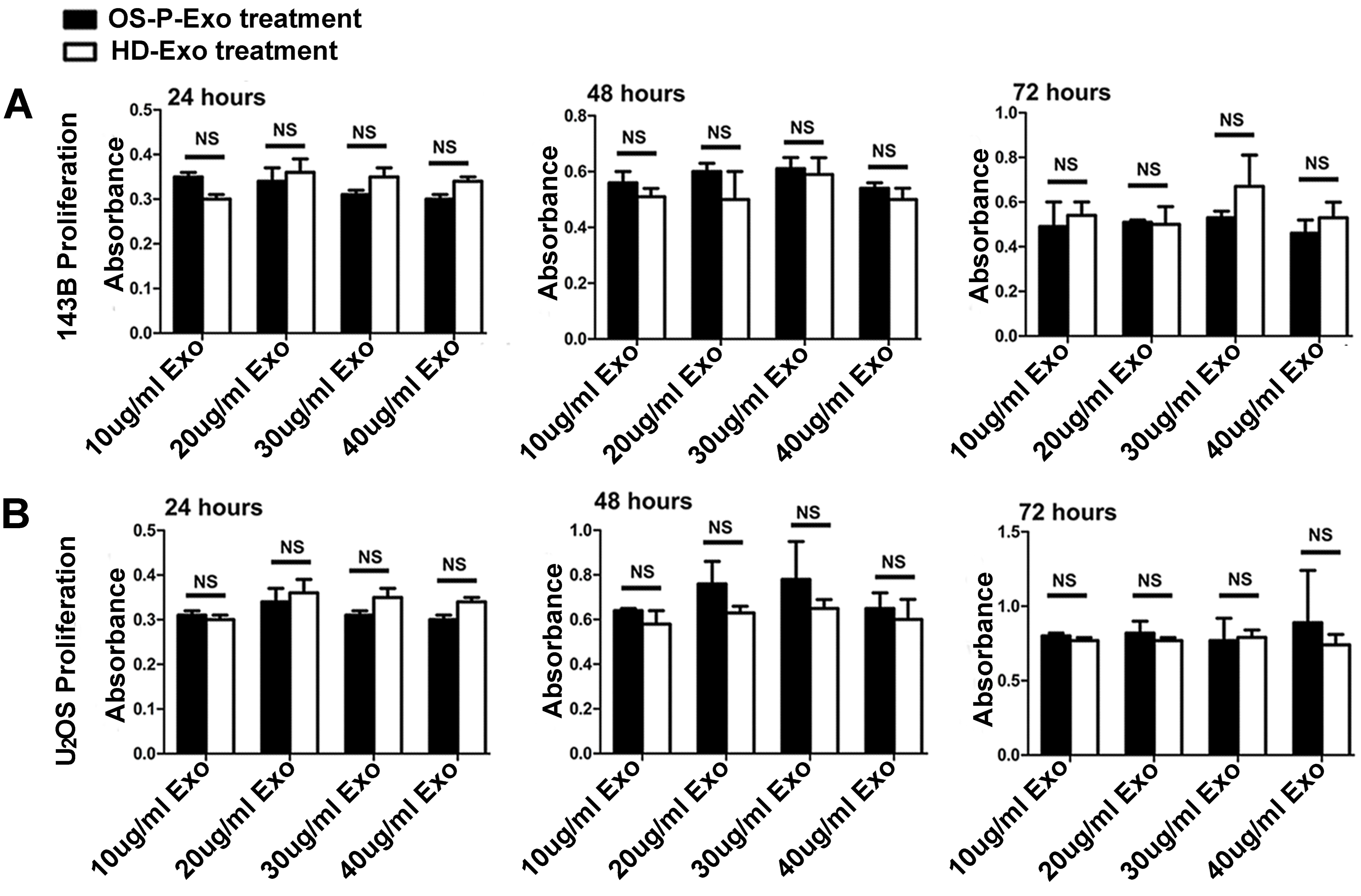

Supplement: Supplementary file 2 — Additional file 2: Figure-1. Treatment of 143B and U2OS cells for 24, 48 and 72 hours using different concentration of Sr-exosomes derived from OS patient and healthy donor. [file 12951_2020_710_MOESM2_ESM.tif]

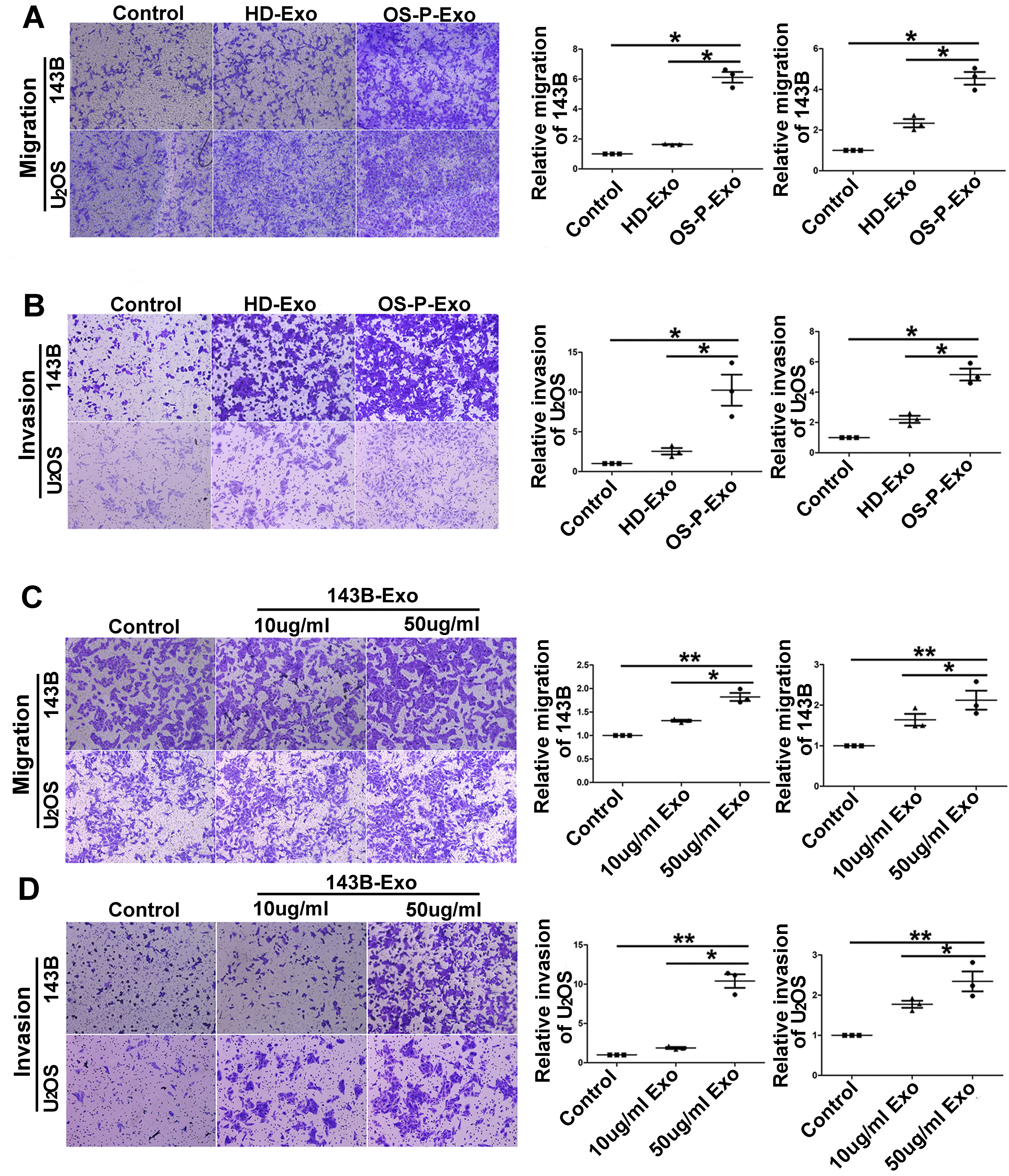

Supplement: Supplementary file 3 — Additional file 3: Figure-2 A-B. Effect of exosomes isolated from serum of OS patient and healthy donor on the migration and invision of 143B and U2OS in vitro; C-D. Effect of different concentration of exosomes isolated from 143B cell on the migration and invision of 143B and U2OS in vitro. [file 12951_2020_710_MOESM3_ESM.tif]

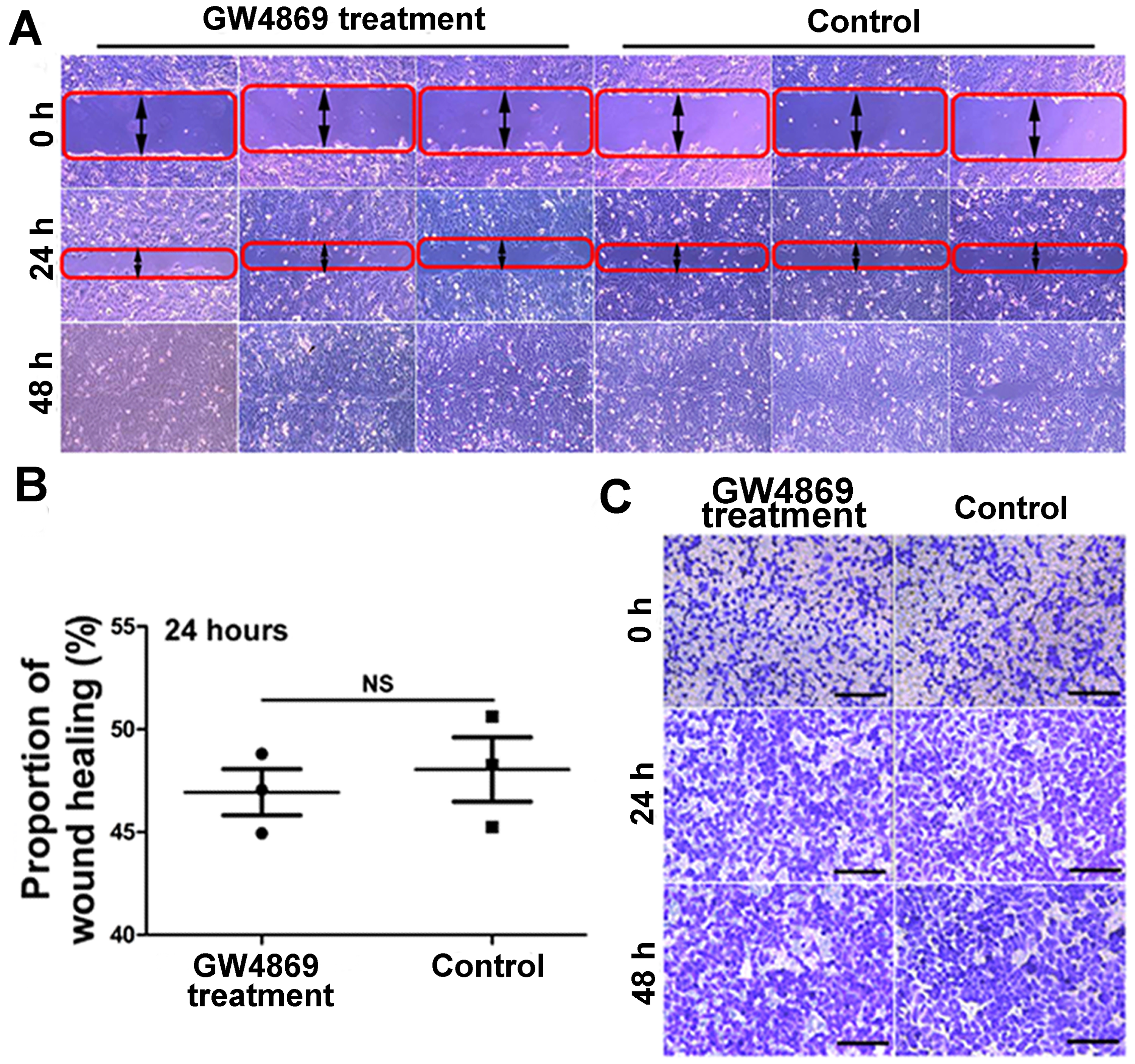

Supplement: Supplementary file 4 — Additional file 4: Figure-3 A-B. Effect of the small molecule of exosome secretion inhibitor GW4869, on the wound healing of 143B cells in vitro, indicating no influence of GW4869 on the wound healing; C. no influence of GW4869 on the migration of 143B cells in vitro. [file 12951_2020_710_MOESM4_ESM.tif]
